# Frugal Self-Optimization Mechanisms for Edge–Cloud Continuum

**DOI:** 10.3390/s25216556

**Published:** 2025-10-24

**Authors:** Zofia Wrona, Katarzyna Wasielewska-Michniewska, Maria Ganzha, Marcin Paprzycki, Yutaka Watanobe

**Affiliations:** 1 Faculty of Mathematics and Information Science, Warsaw University of Technology, 00-662 Warsaw, Poland; 2 Systems Research Institute, Polish Academy of Sciences, 01-447 Warsaw, Poland; katarzyna.wasielewska@ibspan.waw.pl (K.W.-M.); marcin.paprzycki@ibspan.waw.pl (M.P.); 3 Department of Computer Science and Engineering, University of Aizu, Aizu-Wakamatsu 965-8580, Japan; yutaka@u-aizu.ac.jp

**Keywords:** cloud-edge continuum, self-*, frugality, anomaly detection, adaptation, sampling

## Abstract

The increasing complexity of the Edge–Cloud Continuum (ECC), driven by the rapid expansion of the Internet of Things (IoT) and data-intensive applications, necessitates implementing innovative methods for automated and efficient system management. In this context, recent studies focused on the utilization of self-* capabilities that can be used to enhance system autonomy and increase operational proactiveness. Separately, anomaly detection and adaptive sampling techniques have been explored to optimize data transmission and improve systems’ reliability. The integration of those techniques within a single, lightweight, and extendable self-optimization module is the main subject of this contribution. The module was designed to be well suited for distributed systems, composed of highly resource-constrained operational devices (e.g., wearable health monitors, IoT sensors in vehicles, etc.), where it can be utilized to self-adjust data monitoring and enhance the resilience of critical processes. The focus is put on the implementation of two core mechanisms, derived from the current state-of-the-art: (1) density-based anomaly detection in real-time resource utilization data streams, and (2) a dynamic adaptive sampling technique, which employs Probabilistic Exponential Weighted Moving Average. The performance of the proposed module was validated using both synthetic and real-world datasets, which included a sample collected from the target infrastructure. The main goal of the experiments was to showcase the effectiveness of the implemented techniques in different, close to real-life scenarios. Moreover, the results of the performed experiments were compared with other state-of-the-art algorithms in order to examine their advantages and inherent limitations. With the emphasis put on frugality and real-time operation, this contribution offers a novel perspective on resource-aware autonomic optimization for next-generation ECC.

## 1. Introduction

With the ongoing expansion of the Internet of Things (IoT) and the increasing demand for data-intensive applications that involve capturing, analyzing, and exchanging large amounts of data, which are being processed and used for decision-making, the concept of Edge–Cloud Continuum (ECC) has gained significant popularity [1]. Its use cases span several domains, in which heterogeneous resources are being integrated to provide complex solutions. These include, among others, smart homes and cities [2,3], autonomous vehicles [4], smart manufacturing [5], energy management [6,7], or agriculture [8]. As outlined in [9], ECC can be understood as a complex distributed infrastructure that encompasses a wide variety of technologies and interconnected devices, seamlessly integrating the edge (where data sources and actuators are located) and the cloud (infrastructure with computational resources). Through such an integration, ECC aims to harness the combined benefits of both paradigms, while addressing their inherent limitations [10]. In particular, edge devices located in close proximity to the data sensors and actuators are often utilized for time-critical or privacy-sensitive applications, which require minimal latency [11]. On the other hand, cloud resources provide the ability to perform computationally intensive tasks that exceed the capabilities of resource-restricted devices. With dynamic resource provisioning and scalability, cloud solutions can also better accommodate long-running workflows, or those executed over complex interconnected architectures [12,13].

However, despite the aforementioned benefits, ECC also faces a number of challenges [1,14]. (1) The heterogeneity of computational resources, system topologies, and network connectivity schemes hinders the possibility of applying unified orchestration methods, making system management particularly difficult [15,16]. This encompasses not only aspects of resource provisioning, task offloading, or scheduling, but also (2) mechanisms for data management [17]. Achieving the data consensus, in ECC with distributed data sources, requires advanced mechanisms for synchronization, transmission policies, and control over access rights. Here, an additional level of complexity arises from (3) the need to perform all these operations on real-time streaming data. Moreover, (4) the processing of data on, often, resource-limited edge devices requires the use of algorithms with a low computational overhead, occupying little storage [18]. Developing such frugal algorithms typically involves finding a reasonable compromise between efficiency and effectiveness. Finally, (5) the dynamic and distributed nature of the environment impedes the detection of potential errors, which can hinder the system’s stability, resulting in temporary service interruptions, performance deterioration, and breaches of Service Level Agreements (SLA)s [19,20].

Therefore, attaining ECC-based systems reliability requires the incorporation of smart mechanisms providing fault detection and enabling system adaptability to the ever-changing conditions. In order to achieve these objectives, in a possibly automated and feasible manner, self-* mechanisms [21] have become the focus of recent research studies. These mechanisms aim to enhance the system’s autonomy, allowing it to initiate decisions and adaptations with little to no human intervention. In the survey [22], the authors presented a taxonomy that distinguished nine different types of self-*, of which the primary four are: (1) *self-healing*, (2) *self-protection*, (3) *self-configuration* and (4) *self-optimization*. Among these, the *self-optimization* constitutes the focus of this contribution.

Typically, *self-optimization* is understood as a property of the system that not only recognizes the need for its adaptations, in the case of violation of its high-level objectives, but also attempts to improve the system’s performance in its stable state. This makes it particularly important for complex ECC infrastructures that require automation of complex processes. Interestingly, as identified in [22], among other self-* modules, *self-optimization* was among the ones mentioned the least number of times in the existing literature. This fact can be attributed to several factors, including (1) difficulty in specifying necessary key performance indicators, (2) vague definition of the *self-optimization* scope, or (3) the need to incorporate domain-specific expert knowledge. Moreover, the existing works target mostly cloud applications, relying on non-frugal algorithms that either require substantial computational power (e.g., those based on deep neural networks) or storage (e.g., making decisions based on historical data). As a result, they cannot be considered suitable for use on edge nodes that are highly resource-constrained. Interestingly enough, even though not reflected in the literature, *self-optimization* close to the edge seems to be a very attractive solution, as by being deployed in the proximity of data sources, it facilitates fast reactions and adaptations.

In this context, the research in edge self-optimization still exhibits several unresolved gaps. In terms of the applicability domains, the majority of the studies discuss *self-optimization* in the context of adaptable resource management, such as resource allocation, auto-scaling, replica control, and task migration. Apart from the use cases of adaptable workload prediction [23], the *self-optimization* does not appear directly in the context of data dissemination, which pertains to a significant concern in ECC, since frequent transmissions of large data volumes involve considerable energy consumption. Additionally, the current studies mainly focus on *how* to execute adaptation rather than *when* the adaptation should be initiated (e.g., when the potential abnormality appears in the system). Consequently, the timely identification of opportunities for adaptation remains insufficiently explored. Moreover, even though some of these works address edge self-optimization, they still require resource-intensive operations to be offloaded to the cloud [24], making them reliant on external high-performance hardware.

The aforementioned gaps in the existing *self-optimization* literature have been partially addressed in separate studies devoted to anomaly detection [25] and adaptive sampling [26,27]. Consequently, this work will examine how to employ both of these techniques within the *self-optimization* module.

In particular, this contribution presents a frugal *self-optimization* module, in which statistical-based techniques are used to identify potential adaptation opportunities. The article describes two principal roles of the module:

1. **Anomaly detection**—detection of potential resource utilization abnormalities, which was achieved through density-based analysis of real-time data streams collected from computational nodes
2. **Adaptive sampling**—estimation of the optimal sampling period for the monitoring of resource utilization and power consumption, obtained by analyzing the changes in data distribution with Probabilistic Exponential Moving Average (PEWMA)

In the context of ECC, such a module is particularly important, since it enables automated decision-making directly at the resource-constrained edge devices, achieving optimizations in (soft) real-time. By adjusting sampling rates autonomously, the module ensures that data are monitored and (in many cases) transmitted only when necessary. This, in turn, can reduce energy consumption and lessen network congestion. Moreover, applying the anomaly detection mechanisms can support not only fault prevention and the security of ECC but may also serve as a trigger for autonomous adaptations that could help preserve the computational resources.

For example, consider a remote health monitoring system, where patients wear electrocardiogram (ECG) smart watches that measure their vital signs (e.g., heart rate). These ECGs serve as sensors that send the collected data to patients’ smartphones (edge devices), which are responsible for making immediate, critical decisions locally and forwarding relevant information to the healthcare provider’s cloud for more extensive, long-term analysis. In this scenario, the proposed *self-optimization* module could be utilized to automate the collection of patients’ vital signs and recognize potential critical health events. Specifically, by lowering the data collection frequency during long periods of physiological stability (e.g., sleeping or resting), adaptive sampling could prolong the battery life of wearable ECGs. Simultaneously, the anomaly detection mechanism could streamline detection of irregularities (e.g., in a heart rhythm) that could trigger alerts requiring immediate action (e.g., increasing the ECG sampling rate and sending a signal to healthcare professionals). From the perspective of system resilience, anomaly detection could also be used to monitor sensor resources (e.g., temperature) to detect their malfunction (e.g., overheating) and initiate autonomous adaptations (e.g., by switching off non-critical processing).

However, in order to make the *self-optimization* module deployable on the edge, it was necessary to propose an approach that would address the limitations of resource-constrained edge devices. Specifically, the selection of algorithms was guided by factors such as (1) computational efficiency and (2) frugality, understood in the context of both utilized resources and processed data. Therefore, this contribution also provides a structured perspective on the approach to real-time optimization in the context of edge computing. Moreover, the novelty of the proposed solution lies in its modular architecture, which (1) enables the dynamic extension of models to accommodate new data metrics (e.g., network throughput, sensor battery level) and (2) facilitates Human-In-The-Loop (HiTL) interaction for model parameter adjustment (e.g., acceptable estimation imprecision).

The conceptualized *self-optimization* module was implemented as part of the Horizon 2020 European research project aerOS (https://aeros-project.eu/, access date: 24 October 2025). aerOS is a platform-agnostic, intelligent meta-operating system for the edge-IoT-Cloud continuum. It is composed of a wide variety of heterogeneous computational nodes called Infrastructure Elements (IE)s, which operate on different types of devices ranging from embedded microcontrollers to cloud servers that can be potentially distributed between different client domains. In this contribution, aerOS was used as a primary testbed for validating the *self-optimization* module. Nevertheless, it should be noted that the module is also suitable for other ECC applications.

The performance of the developed module has been evaluated using synthetic and real-world complex datasets. In particular, the anomaly detection model was assessed using (1) the Numenta Anomaly Benchmark (NAB) [28] on the synthetic time-series data and (2) a snapshot of CPU utilization monitored in the aerOS continuum. On the other hand, the effectiveness of sampling period estimation was evaluated on both of the aforementioned datasets and, additionally, on RainMon CPU utilization traces (https://github.com/mrcaps/rainmon/blob/master/data/README.md, access date: 24 October 2025). Finally, the achieved results were compared with selected frugal state-of-the-art approaches, including Adaptive Window-Based Sampling (AWBS) [29] and User-Driven Adaptive Sampling Algorithm (UDASA) [30].

The remainder of the article is structured as follows. Section 2 contextualized the implemented approach by reviewing existing studies on the topics of *self-optimization*, adaptive sampling, and real-time anomaly detection. Next, Section 3 describes the developed *self-optimization* module, discussing its main assumptions and requirements, as well as explaining the details of the proposed design. It is followed by Section 4, which presents the selected test cases and discusses the achieved results. Finally, Section 6 summarizes the findings of the study and indicates future research directions.

## 2. Related Works

The *self-optimization* has been addressed in both cloud and edge–cloud infrastructure contexts. However, it is difficult to find recent works that mention this topic directly. The ones that are doing so target mostly the aspect of resource optimization, whereas the concept of “resource” is often perceived from the cloud perspective, rather than the edge (or jointly).

Among others, this can be observed in one of the recent surveys focused on self-* capabilities in ECC [22]. Out of 77 papers that were selected by the authors, only seven of them address the *self-optimization*. Furthermore, the vast majority of these articles were published before 2020. This observation may not necessarily imply a decline of interest in autonomous optimization for the edge and the cloud. Instead, it emphasizes a larger issue, of a vague specification of the scope of different self-* modules.

In particular, there is no uniform definition that establishes the range of impact and the subject areas addressed by different self-*. This is of particular concern when it comes to non-principal modules (i.e., those outside of the original IBM autonomic computing manifesto [21]). For example, the distinction between *self-adaptation*, *self-optimization*, *self-scaling*, *self-configuration*, and *self-learning* often depends on the approach and background of individual researchers and/or the particular application context. As a result, in existing research works, these terms are used to describe overlapping mechanisms. Moreover, lack of clarity regarding the definition of *self-optimization* may also result from the broad understanding of the concept of optimization within systems. In the works identified throughout this section, the *self-optimization* pertained to both (1) a separate module overseeing the infrastructure state and suggesting adaptations to its components, but also (2) an inherent part of one of the internal algorithms, facilitating its autonomy.

For example, in [31], the authors considered *self-optimization* from the perspective of energy efficiency. In particular, they introduced an energy-efficient mechanism for cloud resource allocation in virtual data centers. The design of the proposed approach followed a commonly adopted MAPE-K model [21]. The information about energy consumption was collected by the monitoring module and then passed to the analysis component, responsible for triggering adaptation alerts and constructing corresponding plans. The authors considered two types of adaptations: (1) resource re-allocation, initiated whenever the estimated value of energy consumption fell outside of the specified threshold, and (2) allocation of new resources from the reserve resource pool, when the execution requirements were violated. Here, it should be noted that the energy consumption of cloud resources was recursively computed using past statistics stored in the database after each resource provisioning. Consequently, this solution may not be suitable for edge devices since it may require substantial storage capacity.

In the context of resource management, the authors of [32] proposed an autonomic resource management system with a main focus on energy-saving and Quality of Service (QoS). The system was operating in three different modes: (1) prioritizing energy-saving, (2) prioritizing QoS, and (3) balancing between energy-saving and QoS. The authors described the *self-optimization* as the ability of the system to switch between proposed modes autonomously. However, similarly to the previous work, the appropriate mode was selected by applying a set of threshold-based rules to the predicted application’s workload, which was derived from the past resource utilization history.

A different approach to *self-optimization* was introduced in [23]. There, the generic workload prediction was applied. The authors utilized Long Short-Term Memory (LSTM) to identify workflow patterns. In this case, the *self-optimization* was perceived through the fine-tuning of model parameters with the use of Bayesian Optimization. Nevertheless, the reliance on computationally intensive models and the limited scalability of the optimization approach hinder the applicability of this method in edge scenarios.

Contrary to previous examples, a perspective on *self-optimization*, dedicated directly to the application on the edge, was outlined in [33]. Here, an infrastructure was composed of a federation of edge mini-clouds (EMC) supervising the devices with limited resources. The goal of *self-optimization* was to limit the number of redundant instances of running applications and, as such, optimize the energy utilization. It was achieved by comparing shared applications between neighboring EMCs and identifying users who could be transferred to a single one. Although this approach is computationally efficient and, therefore, well suited for edge applications, it is highly domain-specific since it assumes a very distinct continuum structure composed of mini-clouds. Accordingly, adjusting it to the more generalized case of ECC may be non-trivial.

When evaluating the *self-optimization* solutions, one must take into account these studies, in different sub-domains, that do not explicitly use self-* terminology. In the context of this work, two such domains were analyzed: (1) adaptive sampling and (2) anomaly detection. These domains were selected as they target two important concerns of ECC: (1) excessive energy consumption associated with frequent dissemination of large data volumes, and (2) prevention of malfunctions in ECC components.

In light of the specificity of edge devices, the selection of literature reviewed in the next sub-sections was guided by the following criteria: (1) frugality of the algorithms, (2) their time efficiency and ability to operate in (soft) real-time, as well as (3) suitability to work on streaming data. Let us begin the overview with frugal adaptive sampling techniques.

### 2.1. Frugal Algorithms for Adaptive Sampling

Adaptive sampling is a technique that dynamically adjusts the sampling rate to the evolution of the current metric stream [34]. It aims to (1) reduce the monitoring frequency in states when the differences in subsequent observations are negligible, or (2) increase it when the observations begin to fluctuate. In such a manner, it aids in reducing resources and energy wasted with redundant data dissemination, while at the same time ensuring that monitoring components are alerted to unexpected abruptions.

In the context of edge applications, which require real-time processing and are subject to computational constraints, the most suitable adaptive sampling algorithms employ simple statistical-based models.

In [29], the authors introduced AWBS, a window-based adaptive sampling technique that uses simple moving averages. Specifically, the moving averages were computed over the subsequent windows of incoming time-series data, while their percentage difference was used as a measure of the metric stream evolution. The window size increased if the difference remained within the threshold or decreased otherwise. Although the proposed algorithm is lightweight, it may oversimplify the complex data dynamics. For instance, it should be noted that the usage of simple moving averages puts equivalent weights on all considered observations. Thus, it may overlook intermediate changes in metric stream distribution.

A similar approach that facilitates dynamic sliding windows was proposed in ApproxECIoT [35], where the stratification sampling was considered. Here, the size of a dynamic sliding window (stratum) was defined with an adaptable parameter, controlled by the data stream rate and the availability of resources. The adjustment to the stratum size was computed using Neyman allocation, with the use of a standard deviation of the current sample. Even though the proposed method appears feasible, it is highly specialized for stratification sampling. Specifically, it requires a prior division of the data stream into subsequent types. This is achieved using a dedicated Apache Flume framework that combines data streams from different devices. As such, it may not be applicable to cases where, for instance, data are collected from a single device only (i.e., no need for the division into different types) or when it is highly heterogeneous and it is not possible to form coherent strata.

The authors of UDASA [30] proposed a more generalized approach, where instead of the sampling window, the subject of an adaptation was the frequency. In particular, the method facilitated an enhanced sigmoid function that modified the sampling frequency based on the Median Absolute Deviation (MAD) of the most recently sensed data. The size of the collected data was specified using the sliding window, while the range for frequency adaptation was controlled through a user-defined saving level parameter. Despite the fact that MAD is robust to outliers, it is less sensitive to sudden fluctuations. Hence, it may result in omitting individual data spikes or handling them with significant delays. Consequently, such an approach may not be the best suited to time-critical applications.

As an alternative, an algorithm that performs computations on individual observations has been introduced in the Adaptive Monitoring Framework (AdaM) [34]. In this study, the current evolution of the metric stream was estimated using the PEWMA following a Gaussian signal distribution. The sampling period formula was derived from the difference between the estimated and observed stream deviations, and then compared with the target confidence parameter. This algorithm is indeed lightweight and extremely memory efficient. However, it should be noted that despite the author’s claim of a small number of parameters, six of them need to be set by a system operator, which can be considered to be one of the drawbacks of this approach.

There are also adaptive sampling studies that apply more advanced statistical techniques, e.g., linear regression. In [36], the authors proposed a method consisting of three steps: (1) acquisition process, (2) linear fitting, and (3) adaptive sampling strategy. As part of the presented approach, a linear regression model was constructed using time-series sampling data collected from the sensors. The model was trained on the indicated number of samples, and then the median jitter sum was used to represent the changing trend of the data stream. One of the significant limitations of this work is the assumption of the linearity of the short-term data, which may not be applicable in the majority of real-world applications. Furthermore, periodic storage of relevant samples in the database consumes extra storage and computational resources. This, in turn, may impede the scalability of the solution (e.g., not all restricted devices can store large quantities of data samples). Obviously, data could be sent to the “central repository”, but this defeats the purpose of performing analysis at the edge and brings back problems with using this approach in time-critical applications.

Separately, in [37], the adaptation of the sampling period was addressed with the use of a Kalman filter. In particular, the authors proposed the methodology of an adjustable sampling interval for the water burst detection. The water bursts were detected by calculating the flow residuals and using low-pass filtering. The adaptation of the sampling period was carried out when the computed values violated the predefined thresholds. A significant limitation of this work is its assumption that there is a periodic similarity between past and present data patterns. Specifically, the authors argued that the data patterns for the current week are equivalent to those of the previous week. Moreover, they argued that the missing historical data could be sufficiently estimated using Lagrange’s interpolation. Following such an assumption neglects the possibility of the occurrence of unpredictable or abnormal events, potentially degrading the quality of Kalman predictions.

All the analyzed adaptive sampling approaches have their individual benefits and limitations. The summary of reviewed algorithms is presented in Table 1.

As can be observed, the majority of adaptive sampling algorithms target the sampling period while utilizing simple statistical measures (e.g., moving average, median, and standard deviation). Most of them are general-purpose and, hence, can be applied to a wide variety of ECC applications. Moreover, it is noticeable that almost all algorithms perform computations over the batch of data, rather than individual observations. It should be noted that these algorithms may not be the most suitable for highly critical applications, as they may fail to detect sudden data spikes (e.g., in the Kalman filter, abrupt changes can be potentially lost due to smoothing). Additionally, within this group, some of the algorithms require supplemental data storage for historical information to estimate the evolution of the metric stream.

Among the analyzed works, the AdaM adaptive sampling technique [34] was determined to be the most suitable for the *self-optimization* module. In contrast to the remaining methods, this algorithm relies only on the metrics related to the previous observation, hence requiring a minimal amount of storage. Furthermore, by employing a probabilistic approach, the technique can rapidly accommodate spikes that are likely to occur in edge computing scenarios. These spikes, however, do not dominate future updates, since the probability does not depend on historical observations. Hence, AdaM can be suitable for rapidly fluctuating streams. A notable method’s limitation lies in several model parameters. However, this concern was mitigated on the side of *self-optimization* design, by integrating the HiTL interfaces (as described in Section 3.4).

Presented adaptive sampling algorithms enhance data monitoring across various ECC components by optimizing the energy used for data transmission. While such online monitoring helps maintain awareness of component states, it does not provide direct measures for detecting sudden abnormal behaviors. This requires adopting dedicated anomaly detection techniques, which are analyzed in the following subsection.

### 2.2. Frugal Algorithms for Real-Time Anomaly Detection

According to [38], an anomaly is *an observation, which deviates so much from other observations as to arouse suspicions that it was generated by a different mechanism*. Consequently, anomaly detection is a data mining technique that aims to identify such abnormalities within data. One may distinguish two main types of anomaly detection approaches: contextual or point-based. To illustrate them, let us consider an example of network traffic monitoring. In point-based anomaly detection, any singular abrupt increase in network traffic may be considered anomalous, potentially indicating a security breach such as a DDoS attack. However, in the case of contextual anomaly detection, which often incorporates prior knowledge (e.g., user behavior patterns), the same observation may be classified as normal if it appears within a recurring period of increased user activity.

Both of these approaches have been extensively studied in recent years. For example, in the case of contextual anomaly detection, authors of LightESD [39] introduced an approach that is based on the extraction of periodograms and residuals. In particular, the seasonality is obtained by computing the periodograms using Welch’s method. Then, the technique uses decomposition to extract and remove the trend and seasonality components in order to obtain the residual. Finally, the anomalies are detected by performing a modified version of the Extreme Studentized Deviate (ESD) test. As indicated by the authors, the major limitation of the proposed approach is that the method requires a batch of data in order to learn the underlying patterns, which may not be available in the case of all applications.

Another approach to contextual anomaly detection was proposed in [40], where the authors used binary classification and hypothesis testing. At the first offline stage, the normal data were divided into a predefined number of subsets, where each subset was a subject of k-means clustering. The union of all clusters was regarded as a region of normal data. Hence, a new observation was considered anomalous whenever it did not fit into any of the clusters. This anomaly classification was used to obtain a binary data stream. In the last step, authors employed either of two methods for anomaly pattern detection: (1) APD-HT, with hypothesis testing, or (2) APD-CC, with control charts. Although this technique is lightweight, while working on the streaming data, it relies on the offline model training step. It is assumed that a training dataset contains only normal observations, which is also a significant limitation, especially in systems that lack prior domain data. Moreover, relying on the offline pre-trained model does not accommodate the evolution of the data stream.

The latter problem was addressed in [41], where another Contextual Anomaly Detection (CAD) framework was proposed. Here, the authors described a semi-online anomaly detection method for contextual anomalies in the energy consumption data streams. In order to predict short-term energy consumption, the framework used an LSTM. The anomalies were identified by comparing the predicted and actual consumption values with errors modeled using a rolling normal distribution. Moreover, the authors employed a CAD-D3 method for the concept drift detection, which triggered the retraining of the prediction model. Although the authors mentioned the IoT applications, the method is arguably too computationally intensive to be employed on edge devices. Moreover, it relies on several hyperparameters, tuning of which may require domain expertise and a number of trials and errors (for each individual deployment).

As an alternative, in [42], the authors introduced PiForest, which modifies a well-known anomaly detection technique, iForest [43], by introducing data pre-processing. In particular, the objective was to overcome iForest’s limitation concerning the usage of substantial storage capacity when processing high-dimensional data. The authors utilized principal component analysis (PCA) for dimensionality reduction. Moreover, to handle streaming data, a sliding window was used. Contrary to the previously described methods, PiForest is not necessarily devoted to contextual anomaly detection. However, tests conducted by the authors suggest its efficacy in such scenarios. Moreover, the technique is suited for deployment on resource-constrained devices. The main disadvantage comes with the hyperparameter sensitivity, as the performance of the algorithm relies on the appropriate setting of the number of trees.

Separately, in the context of point-based anomaly detection, in [44], the density-based technique was proposed. In particular, it was established on the concept of collecting data samples into a cluster. Therefore, upon each new upcoming observation, the recursive density formula was employed to update the information about the sample compactness. Whenever the current density decreased below the mean density, the observation was considered anomalous. The authors employed a non-parametric Cauchy function as a density kernel to decrease the sensitivity to outliers. Moreover, in order to control the transition between normal and anomalous states, additional parameters, including anomaly/normality window and transition thresholds, were introduced. Contrary to offline/semi-online approaches, this algorithm is extremely lightweight and requires no memory to operate. It may, however, not be as effective for the detection of contextual anomalies, as it does not learn complex data patterns.

Another online anomaly detection approach, GAHT, was proposed in [45]. The authors modified the Extremely Fast Decision Tree (EFDT) method [46] to improve its energy efficiency. In particular, the tree growth was optimized through dynamic hyperparameters. The split criteria were dynamically adjusted, based on the distribution of instances at each node. Although the algorithm is rather computationally efficient, it does not perform too well with high-dimensional data, since its time and space complexity depend on the number of attributes. Moreover, contrary to the PiForest, the accuracy of GAHT was not evaluated in the case of contextual anomaly detection.

In order to outline the differences between all summarized methods, their selected characteristics are presented in Table 2.

It can be observed that the techniques dedicated to the detection of contextual anomalies, in the majority, rely on offline or semi-online learning methods. Moreover, they are often associated with the requirement of additional storage capacity. On the other hand, online methods are more often employed for point-based analysis and are better at accommodating concept drifts.

Interestingly, these observations are consistent with the findings of the survey [47], where the authors reviewed the anomaly detection methods for the streaming data. Here, it was noted that the offline methods are the least suitable for real-time applications, due to their computational complexity and a lack of concept drift accommodation. The semi-online methods were deemed to perform better, but not all of them can be utilized on resource-limited devices, as some require additional storage for historical data. Therefore, among those categories, online methods were considered the most appropriate for ECC.

All of the aforementioned conclusions were taken into account when selecting the anomaly detection approach for the *self-optimization* module. In particular, it was decided that the module will implement a density-based approach [44]. Among all point-based methods, that approach was considered the most computationally efficient and generalizable. It does not require extensive parameter tuning and is the most explainable, enabling the easy engagement of HiTL. Moreover, the method itself is memoryless, since its calculations are based solely on the mean and scalar product, making it suitable to be deployed on resource-restricted devices.

The selected adaptive sampling and anomaly detection methods served as the core components of the implemented *self-optimization* module, which is described in detail in the following section.

## 3. Implemented Approach

The *self-optimization* module belongs to the collection of nine self-* modules available to be deployed in the aerOS. Its primary role is to analyze the current state of the computational resources and to suggest potential adaptations that could lead to broadly understood efficiency improvements. In order to achieve this objective, *self-optimization* employs two mechanisms: (1) adaptive sampling, which is used to autonomously control the frequency of data monitoring, and (2) anomaly detection that aims to identify and alert about potential abnormalities in the resource utilization. Before proceeding to the in-depth description of each of these mechanisms, let us first discuss the requirements that guided their design. It should be emphasized that although these requirements were formulated in accordance with the particular infrastructure (e.g., aerOS) in which the module was tested, they are generalizable to other scenarios as well, making the proposed design versatile across different applications.

### 3.1. Requirements

The requirements were derived based on the specificity of the environment in which the *self-optimization* module is intended to operate. Therefore, prior to listing them, it is necessary to outline the main characteristics of aerOS.

Let us recall that aerOS is an intelligent meta-operating system for the edge-IoT-Cloud continuum. The operations in aerOS are performed on the computational nodes called IEs. As these may differ in terms of available resources or their internal architecture, no assumptions should (and can) be made regarding their computational capabilities. Moreover, it should be noted that tasks executed on each IE are treated as “black boxes”, meaning that the only attributes known to the system are their execution requirements.

In order to improve the management and performance of the entire infrastructure, the aerOS provides a set of mandatory and auxiliary services deployable on individual IEs. Among these services are nine self-* modules [22], which aim to enrich IEs with autonomous capabilities. In the context of *self-optimization*, the most important self-* modules are (1) *self-orchestration*, which invokes rule-based mechanisms that autonomously manage IE resources, and (2) *self-awareness*, which continuously monitors the IE state, providing insights on its current resource utilization and power consumption. These modules act as points of interaction with the external environment, from the *self-optimization* perspective. However, they can be substituted with any components that will provide monitoring data and accept the output of the module under discussion.

Taking into account all the aforementioned features of aerOS, the *self-optimization* module was designed according to the following requirements:

1. **Tailored to operate on data streams**: since the *self-optimization* module receives data from the *self-awareness* module, its internal mechanisms must be tailored to work on real-time data streams. Therefore, at this stage, methods that utilize large data batches or process entire datasets were excluded from consideration.
2. **Employ only frugal techniques**: all the implemented algorithms should be computationally efficient and require a minimal amount of storage. This requirement was established to enable deploying *self-optimization* on a wide variety of IEs, including those operating on small edge devices. Consequently, no additional storage for historical data was considered, which eliminated the possibility of implementing some of the more advanced analytical algorithms. However, it should be stressed that this was a design decision rooted in in-depth analysis of pilots, guiding scenarios, and use cases of aerOS and other real-life-anchored projects dealing with ECC.
3. **Facilitate modular design**: all internal parts of the *self-optimization* should be seamlessly extendable to enable accommodating new requirements, or analyzing new types of monitoring data. Therefore, *self-optimization* should employ external interfaces that would facilitate the interaction with human operators, or other components of the aerOS continuum. Fulfilling this requirement will allow for generic adaptability of the module since data/metrics to be analyzed may be deployment-specific.

These requirements dictated the overall architectural design of the *self-optimization* module, as well as influenced the selection of its underlying algorithms, which are described throughout the following sub-sections.

### 3.2. Self-Optimization Architecture

**Figure 1:**
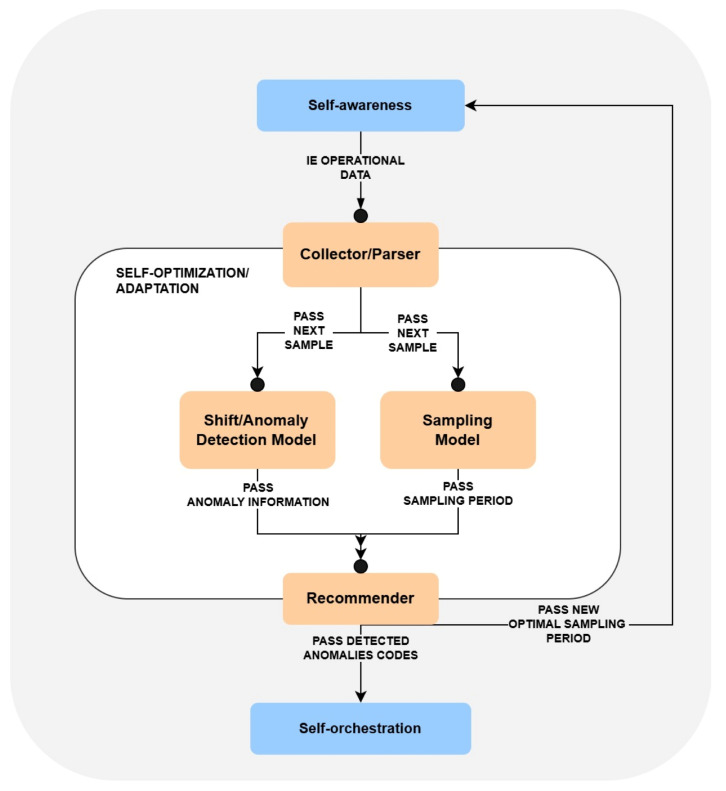
Architecture of *self-optimization* module.

The high-level architecture of the *self-optimization* is depicted in Figure 1. It illustrates the core parts of the module, which are: (1) *Collector/Parser*, (2) *Shift/Anomaly Detection Model*, (3) *Sampling Model* and (4) *Recommender*. Let us now describe the responsibilities of each of these components and their role in processing the received monitoring data.

The *Collector/Parser* is the component that receives the hardware state of IE sent periodically by the *self-awareness* module. It can be thought of as a gateway that collects raw input and maps it into a format that can later be used for further analysis. In particular, the data sent by the *self-awareness* module consists of a set of different metrics, among which not all are relevant from the perspective of the implemented analytical models (e.g., IE location, CPU architecture, or trust score).

Therefore, it is the responsibility of *Collector/Parser* to extract the required metrics. Currently, these include the CPU cores, RAM, and disk utilization, as well as the power consumption. Here, it should be noted that these metrics are stored in the form of key-value pairs; hence, extending their scope in the future would be easily possible.

After formatting the IE data, *Collector/Parser* forwards it to two analytical components— *Shift/Anomaly Detection Model* and *Sampling Model*, which are responsible for the identification of potential adaptation possibilities.

In particular, the role of the *Shift/Anomaly Detection Model* is to determine whether the value of any of the considered utilization metrics should be classified as anomalous. Separately, *Sampling Model* estimates the most appropriate frequency for monitoring, of the IE state, in *self-awareness*. The process of detecting the anomalies is further described in Section 3.3, while the applied adaptive sampling approach is discussed in Section 3.4. The results of both analyses are passed to the last *self-optimization* component, which is the *Recommender*.

*Recommender* is responsible for forwarding the suggested adaptations to the associated self-* modules. Specifically, its functionality is organized into two distinct flows. (1) Upon receiving the proposed most optimal sampling period from *Sampling Model*, *Recommender* forwards that information to the *self-awareness* so that it can adjust the monitoring frequency. Moreover, (2) after obtaining the details about the detected anomalies from *Shift/Anomaly Detection Model*, it maps each anomaly type into a corresponding unique code and sends them to *self-orchestrator*. In this context, a unique code acts as a particular type of alert (e.g., a sudden increase of CPU usage in a particular infrastructure device can be mapped into a single code 001). The *self-orchestrator* maps each type of alert into a corresponding mitigation/recovery action, represented through a serverless function, and triggers its execution on the common serverless platform OpenFaaS [48]. In aerOS, such functions are predefined by the developers, hence the type of applied mitigation action is specific to the given application domain (e.g., may involve re-allocation of deployed services). Here, let us also note that the communication between *self-optimization* and *self-orchestrator* is highly generic, operating only on the means of a single code. Hence, the proposed *self-optimization* module can be plugged in as a standalone component to any other type of system, which defines its own mechanisms of performing adaptation actions.

Let us observe that the architecture of the *self-optimization* module facilitates the separation of concerns. In fact, it resembles a simplified version of the MAPE-K model. In particular, the data collection and pre-processing in *Collector/Parser* can be viewed as a MAPE-K Monitoring component. The MAPE-K Analysis component is reflected by *Sampling Model* and *Shift/Detection Anomaly Model* since these identify potential adaptation possibilities. Finally, the *Recommender* corresponds to the MAPE-K Planning and Execution. Using such a design supports the high-level modularity of the system, enabling its further extensions.

Since the core components of the analysis are *Shift/Anomaly Detection Model* and *Sampling Model*, let us describe them in further detail.

### 3.3. Shift/Anomaly Detection Model

The primary responsibility of the *Shift/Anomaly Detection Model* is to identify anomalies in the current IE state. Since the representation of the IE state is composed of multiple metrics, there are two main approaches that can be applied in terms of anomaly detection.

The first approach considers the IE state as a whole, meaning that the detected anomalies represent a potential degradation of an overall IE performance but are not associated with any particular (individual) metric of the resource utilization. Application of such an approach requires either scalarizing the IE state to a single value or implementing anomaly detection techniques, which operate on multivariate data. While this may simplify the anomaly detection, requiring only one model to be implemented, it also sacrifices the metric-level interpretability. In particular, since there is no identification of specific anomalous metrics, the root cause of the problem cannot be easily diagnosed. Therefore, determining appropriate mitigation measures may also be very challenging. As an example, an anomaly may be detected due to a memory leak or excessive CPU utilization. These are two separate issues that require completely different handling solutions.

The second approach addresses such cases by detecting anomalies for separate metrics. In particular, the metrics are divided into scalar values based on their type and fed into separate anomaly detection models. Models of this type require individual parameter tuning, which may be viewed as both a disadvantage and an advantage. On the one hand, such an approach requires the adjustment of a vast number of parameters, which may not always be straightforward. However, on the other hand, it may also improve the performance of the models since it allows their behaviors to be adjusted based on the evolution and distribution of individual metric streams. Here, obviously, it is possible that subgroups of parameters can be used for anomaly detection, but further exploring such possibilities is out of the scope of this contribution. Suffices to say that, for obvious reasons, the proposed approach can support anomaly detection based on any subgroup of parameters. All that is needed is to: (a) adjust the *Collector/Parser* to extract the needed parameters, and (b) insert the needed *Shift/Detection Anomaly Model*.

**Figure 2:**
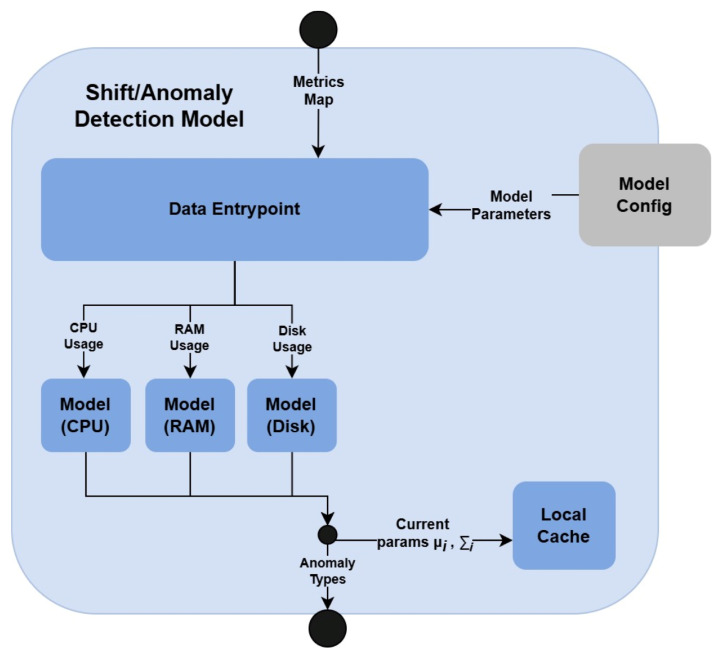
Architecture of Shift/Anomaly Detection Model.

The *Shift/Anomaly Detection Model*, considered in what follows, employs the second approach, which is illustrated on the high-level architecture of the component in Figure 2.

The overall flow of the *Shift/Anomaly Detection Model* is as follows. The IE data are initially sent to the *Data Entrypoint*, which is responsible for extracting values corresponding to each metric type. Let $x_{m}$ denote the observed value of metric *m*. Currently, *Shift/Anomaly Detection Model* takes into account only three types of metrics: (1) CPU utilization $x_{CPU}$, (2) RAM utilization $x_{RAM}$, and (3) disk utilization $x_{disk}$. However, as noted, this scope may be extended in the future, without affecting the remaining parts of the system. For each metric *m*, *Data Entrypoint* fetches the corresponding model parameters from the *Model Config* component. Let $\theta_{m}$ represent the parameter set associated with the model that handles metric *m*. Each pair $\left(x_{m},\theta_{m}\right)$ is subsequently fed into the corresponding model, which applies a density-based anomaly detection algorithm [44]. After finishing the detection process, the anomalies returned by individual models are aggregated and passed to the *Recommender* (see Section 3.2). Moreover, the statistics characterizing the temporal evolution of each metric stream (i.e., sample mean $\mu_{i,m}$ and scalar product $\sum_{i,m}$ of metric *m* at time step *i*) are updated and stored in a lightweight *Local Cache*, to support incremental computations.

Taking this into account, in what follows, a detailed description of the algorithm responsible for anomaly detection is presented.

#### 3.3.1. Density-Based Anomaly Detection

As indicated in Section 2, *Shift/Anomaly Detection Model* implements a density-based point anomaly detection algorithm described in [44]. Specifically, the algorithm represents the evolution of the metric stream through the computation of consecutive sample densities. The notation used in the subsequent algorithm’s description is summarized in Table 3.

The computations begin upon receiving the next observation from the *Collector/Parser* component. In the first step, the sample mean and the scalar product are calculated, using the recursive formulas presented in Equations (1) and (2). It should be noted that both these formulas do not rely on any additional data, apart from the previously computed statistics (i.e., $\mu_{i-1}$, $\sum_{i-1}$). Hence, the AdaM method does not require any additional storage aside from a lightweight local cache. This is important in the case of resource-constrained devices.

(1) $$\mu_{i}=\left\{\begin{array}{cc} \mu_{i-1}+\frac{x_{i}-\mu_{i-1}}{N}, & \mathrm{if}N>0\\ x_{i}, & \mathrm{otherwise} \end{array}\right.$$

(2) $$\sum_{i}=\left\{\begin{array}{cc} \sum_{i-1}+\frac{x_{i}^{2}-\sum_{i-1}}{N}, & \mathrm{if}N>0\\ x_{i}^{2}, & \mathrm{otherwise} \end{array}\right.$$

After updating the sample mean and the scalar product, the obtained values are used in the computation of the sample density. The density formula is inspired by the concept of data clouds (i.e., fuzzy representations of data clusters) introduced in [49]. Here, the Cauchy kernel was used over the data cloud density in order to control the contribution of individual sample points in the estimation, mitigating the impact of outliers. In comparison to other kernels (e.g., Gaussian), Cauchy exhibits heavier tails and slower decay, by which it strongly suppresses the impact of distant samples, while maintaining the contribution of nearby points. As such, it is less sensitive to extreme deviation, anticipating their occurrence, and consequently mitigating their impact on the final estimation. Thus, Cauchy kernels provide greater robustness in scenarios with highly noisy or irregular data. To accommodate the requirements of incremental, stream-based computations, the recursive formula, presented in Equation (3), is applied.

(3) $$D_{i}=\left\{\begin{array}{cc} \frac{1}{1+{\left(x_{i}-\mu_{i}\right)}^{2}+\sum_{i}-\mu_{i}^{2}}, & \mathrm{if}N>0\\ x_{i}, & \mathrm{otherwise} \end{array}\right.$$

The updated density is then used to calculate the mean density, as represented in Equation (4), where the density difference $\Delta D_{i}$ is computed, as formulated in Equation (5).

(4) $${\bar{D}}_{i}=\left\{\begin{array}{cc} \left(D_{i-1}+\frac{\Delta D_{i}}{K}\right)\cdot (1-|\Delta D_{i}\left|\right)+D_{i}\cdot \left|\Delta D_{i}\right|, & \mathrm{if}N>0\\ 1, & \mathrm{otherwise} \end{array}\right.$$

(5) $$\Delta D_{i}=D_{i}-D_{i-1}$$

In the presented equation, the absolute density difference $|\Delta D_{i}|$ serves as a weighting factor, enabling adjustment to the impact of previous and current densities on the estimation. In particular, when the changes in density are minor (i.e., similar observations), the updated mean remains closer to the previous density (i.e., $D_{i-1}+\frac{\Delta D_{i}}{K}$). On the other hand, significant variations favor the contribution of a new mean density (i.e., $D_{i}$). This allows the model to adapt more effectively to large changes, while smoothing out negligible fluctuations.

The mean density is used to control the transition between the normal and the anomalous states. In particular, if the current observation is considered to be normal, then it may switch to the anomalous state according to Equation (6).

(6) $$state=\left\{\begin{array}{cc} anomalous, & \mathrm{if}D_{i}\leq {\bar{D}}_{i}\cdot Th_{anomaly}\wedge K\geq W_{anomaly}\\ normal, & \mathrm{otherwise} \end{array}\right.$$

There are two conditions that must be satisfied for the anomalous state to occur: (1) the current density must be less than the weighted mean density, and (2) the number of consecutive observations *K* that remained in the same state (in this case, potentially anomalous) is above a specified threshold $W_{anomaly}$. Please note that if the first condition (i.e., $D_{i}\leq {\bar{D}}_{i}\cdot Th_{anomaly}$) is fulfilled, then the number *K* will be incremented, but the state will not change to the anomalous until *K* reaches a sufficient size. Therefore, by applying the weighting and the threshold parameters, the sensitivity of the model can be seamlessly adjusted for the specific use cases.

A similar transition also occurs from the anomalous to the normal state. However, since it is not the main subject of this study, more details about this mechanism are omitted, while they can be found in [44].

Upon recognizing the anomalous state, the category of anomaly must be specified. However, it should be noted that, since the algorithm does not rely on historical or labeled data, it cannot learn different types of complex anomalies. Therefore, in *Shift/Anomaly Detection Model*, two anomaly classes were, by default, specified for each type of metric: (1) *INCREASE* (i.e., for density being **above** the mean density) and (2) *DECREASE* (i.e., for density being **below** the mean density). These categories aim to characterize the type of abrupt shifts in resource utilization. However, it should be noted that due to the modular architecture of the module, the anomaly classes can be easily extended or modified, depending on the particular application context.

The quality of anomaly detection is influenced significantly by the parameters of the individual models. In this context, the next subsection describes which parameters were considered and how the human operator can be involved in their dynamic modification.

#### 3.3.2. Model Configuration

The *Shift/Anomaly Detection Model* is composed of several sub-models, each of which requires adjustment of its individual parameters. The density-based anomaly detection parameters encompass: (1) $Th_{anomaly}$, with a value in the interval [0, 1], (2) $Th_{normal}$, with a value in the interval [0, 1], (3) $W_{anomaly}$, with an integer value greater or equate to 1 and (4) $W_{normal}$, with an integer value greater or equate to 1.

All of them are defined accordingly in Table 3. In general, adjusting these parameters is a non-trivial task, which depends on the specific application context. For instance, in time-sensitive applications, such as critical healthcare systems, the detection of anomalies may require a high level of granularity to promptly alert about singular deviations. In such cases, the parameters $W_{anomaly}$ and $W_{normal}$ should be minimized to accommodate transient fluctuations. In the opposite case, in systems characterized by frequent data fluctuations (e.g., high-traffic web services), $W_{anomaly}$ and $W_{normal}$ should be adjusted to larger values to react only to sustained abnormalities.

In this context, it should be noted that the module is not domain-specific and can be utilized in a variety of applications. However, this contributes to the complexity of the parameter fine-tuning, as no universal method exists (and can be applied) to determine their most optimal combination. Furthermore, since the *self-optimization* was designed with modularity in mind, it was considered that *Shift/Anomaly Detection Model* may potentially be extended (in the future) to support new sub-models. Consequently, it was determined that the configuration of parameters must be: (1) *adjustable*—to the specific application context and (2) *extendable*—to support future modifications.

To accommodate these requirements, each of the sub-models was identified by a unique name (e.g., *CPU_MODEL*), allowing it to be associated with an individual set of parameters. These parameters were stored in an adaptable component configuration *Model Config*. Moreover, in order to facilitate the integration of the *Shift/Anomaly Detection Model* into different systems, dedicated interfaces were introduced to support dynamic parameter adjustment by the HiTL.

In particular, the endpoint */anomaly/parameters/{type}* was implemented to provide an on-the-fly parameter modification capability, e.g., for the external human operator. In addition, in order to ensure continuity of system operation, the newly introduced parameters are applied only once the next sample is received, whereas the ongoing analysis proceeds using the previous parameters.

Apart from the *Shift/Anomaly Detection Model*, the second model employed in the *self-optimization* is the *Sampling Model*. Its architecture, along with the utilized method, is described in the subsequent section.

### 3.4. Sampling Model

**Figure 3:**
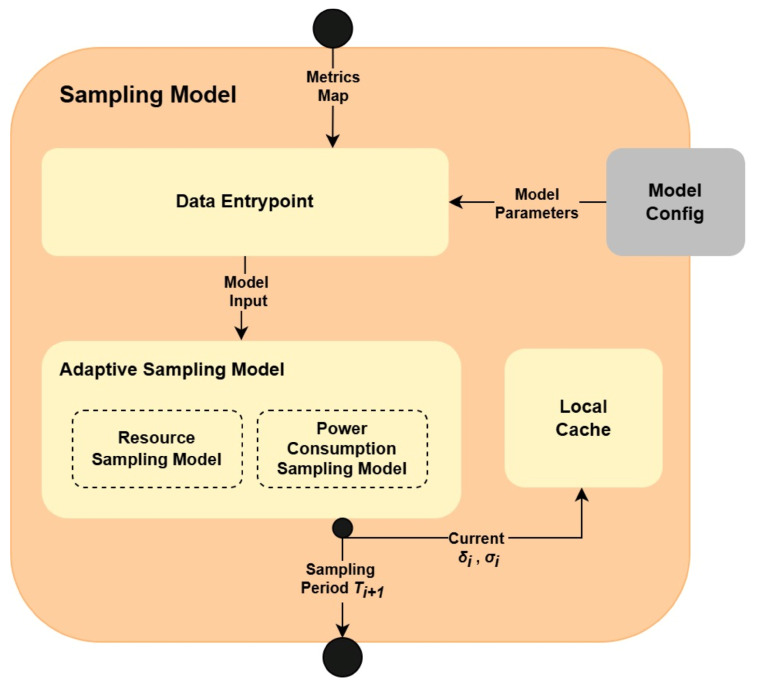
Architecture of Sampling Model.

The *Sampling Model* follows a similar architectural approach as the *Shift/Anomaly Detection Model*. In particular, as illustrated in Figure 3, it is also composed of the (1) *Data Entrypoint*, (2) *Model Config* and (3) *Local Cache*. However, instead of utilizing separate analytical models for each metric type, it contains a single *Adaptive Sampling Model*. This model accepts the entire IE data and handles its processing internally using two sub-models: *Resource Sampling Model* and *Power Consumption Sampling Model*.

The process of handling the IE data in *Sampling Model* is as follows. As with the *Shift/Anomaly Detection Model*, *Sampling Model* receives the information about the current IE state, in *Data Entrypoint*. The *Data Entrypoint* can operate in two modes: (1) processing resource utilization data, and (2) processing power consumption data. Each of these modes is identified by a unique name, being, respectively, *RESOURCE* or *POWER*. The mode that should be adopted by *Data Entrypoint* is specified along the data passed by the *Collector/Parser* component (Section 3.2). Let ${\left[x_{CPU},x_{RAM},x_{disk}\right]}^{T}$ be a vector representing the resource utilization metrics, and let $x_{P}$ be a scalar representing the current power consumption. Instead of splitting the data into different metric types, the main responsibility of *Data Entrypoint* is to select parameters of the corresponding model. These parameters are obtained based on the mode from the *Model Config*. After obtaining the parameters, *Data Enrypoint* creates corresponding pairs: (1) $\left({\left[x_{CPU},x_{RAM},x_{disk}\right]}^{T},\theta_{RESOURCE}\right)$, where $\theta_{RESOURCE}$ denotes the set of parameters of *Resource Sampling Model* for the *RESOURCE* mode, and (2) $\left(x_{P},\theta_{POWER}\right)$, where $\theta_{POWER}$ denotes the set of parameters of the *Power Consumption Sampling Model* for *POWER* mode. These pairs are then passed to the *Adaptive Sampling Model* along with the information about the mode type, so that the component can recognize which internal model should be called. Each of these internal models applies the same AdaM algorithm [34], which, as an output, recommends the optimal sampling frequency. Corollary to the algorithm utilized in *Shift/Anomaly Detection Model*, AdaM is memory efficient. However, it requires storing updated statistics of the metric stream evolution in the lightweight *Local Cache*. This happens as the last step. The next subsection describes the details of the AdaM adaptive sampling algorithm.

#### 3.4.1. AdaM Adaptive Sampling

The original AdaM framework [34] introduces two algorithms: (1) adaptive sampling, and (2) adaptive filtering. In the context of this work, the emphasis is placed only on the adaptive sampling. The core of the algorithm is the estimation of metric stream evolution using PEWMA. Table 4 outlines all the notation used in this section. It should be noted that this section provides an overview of the applied formulas. In order to gain a better understanding of the underlying rationale of the selected statistics, it is advised to see the original article [34].

In order to calculate the PEWMA, it is necessary, first, to compute the distance between the two consecutive observations. In the implemented algorithm, the Manhattan distance formula is used, as presented in Equation (7).

(7) $$\delta_{i}=\left\{\begin{array}{cc} |x_{i}-x_{i-1}|, & \mathrm{if}N>0\\ 0, & \mathrm{otherwise} \end{array}\right.$$

The distance $\delta_{i}$ is utilized in the computation of the probability $P_{i}$, which is a core component of the PEWMA estimation. The probability is computed under the Gaussian distribution, in order to accommodate sudden spikes in the data, while simultaneously controlling their long-term influence. In particular, it prevents singular fluctuations from dominating the overall estimation. The formula for $P_{i}$ is outlined in Equation (8). It should be noted that $P_{i}$ is computed only when N>0, since, in the other case, the default value of PEWMA estimation is used.

(8) $$P_{i}=\frac{1}{\sqrt{2\pi }}\cdot exp\left(-\frac{z_{i}^{2}}{2}\right)$$

Here, $z_{i}$ represents a z-score at time *i*, which is obtained using the current distance $\delta_{i}$, estimated distance $\mu_{i-1}$ (i.e., previous PEWMA estimation), and the estimated moving standard deviation ${\bar{\sigma }}_{i}$ as indicated in Equation (9). It represents the deviation of $\delta_{i}$, relative to the expected data distribution.

(9) $$z=\frac{\delta_{i}-\mu_{i-1}}{{\bar{\sigma }}_{i}}$$

In the formula, ${\bar{\sigma }}_{i}$ corresponds to the moving standard deviation, which can be calculated using the previous PEWMA estimation and the smoothed second moment (i.e., PEWMA of squared deviations; $\nu_{i-1}$). Specifically, it is derived using Equation (10). It should be noted that since all of the formulas are recursive and depend only on previous values, they are well-aligned to the needs of incremental computations.

(10) $$\begin{array}{cc} \nu_{i-1} & =\alpha \cdot \left(1-\beta P_{i-1}\right)\cdot \nu_{i-2}+\left(1-\alpha \cdot \left(1-\beta P_{i-1}\right)\right)\cdot \delta_{i-1}^{2}\\ {\bar{\sigma }}_{i} & =\sqrt{\nu_{i-1}-\mu_{i-1}^{2}} \end{array}$$

Finally, the computed probability $P_{i}$ is substituted in the recursive PEWMA formula, presented in Equation (11).

(11) $$\mu_{i}=\left\{\begin{array}{cc} \alpha \cdot \left(1-\beta P_{i}\right)\cdot \mu_{i-1}+\left(1-\alpha \cdot \left(1-\beta P_{i}\right)\right)\cdot \delta_{i}, & \mathrm{if}N>0\\ \delta_{i}, & \mathrm{otherwise} \end{array}\right.$$

In Equations (10) and (11), the parameters α and β are the weighting factors that control the influence of the previously observed values and the probability.

The anomalies are detected based on the difference between the actual observed standard deviation $\sigma_{i}$ and the standard deviation estimated using PEWMA ${\bar{\sigma }}_{i}$. In particular, Equation (12) presents the formula of a confidence interval, which specifies the degree of certainty of the estimation. It should be noted that the $\sigma_{i}$ may be equal to zero when the consecutive observations are equal. In such a case, the evolution of the metric stream is stable (distance equal to zero). Consequently, the confidence should be maximized in order to increase the sampling period as much as possible, which explains the second condition.

(12) $$c_{i}=\left\{\begin{array}{cc} 1-\frac{|{\bar{\sigma }}_{i}-\sigma_{i}|}{\sigma_{i}}, & \mathrm{if}\sigma_{i}\neq 0\\ 1, & \mathrm{otherwise} \end{array}\right.$$

When the confidence is above the acceptable imprecision (i.e., 1−γ), a new sampling period is proposed, according to Equation (13).

(13) $$T_{i+1}=\left\{\begin{array}{cc} T_{i}+\lambda \cdot \left(1+\frac{c_{i}-\gamma }{c_{i}}\right), & \mathrm{if}c_{i}\geq 1-\gamma \\ T_{min}, & \mathrm{otherwise} \end{array}\right.$$

The formula uses the λ parameter, which allows scaling the estimated sampling period value. It can be particularly useful when applying the AdaM technique in different scenarios that operate on different units of sampling period (e.g., milliseconds, seconds).

The last step, before returning the estimated sampling period, is the verification whether it falls within the boundaries $\left[T_{min},T_{max}\right]$. If the value does not comply with the specified limitations, it is being clamped.

As can be observed, the AdaM algorithm operates on scalar observations. This perfectly accommodates estimating the sampling period in the case of power consumption. However, applying this technique in the case of the *RESOURCE* mode, which uses a vector of values, requires introducing additional modifications.

In particular, the proposed approach is based on the minimization of the sampling period. First, the sampling period is computed separately for each resource utilization metric while using a common set of model parameters. Afterwards, the minimal estimated sampling period is selected in order to account for frequent fluctuations. Taking into account the sampling period of relatively stable metrics (associated with the greater values of the sampling period) would not be feasible, since the substantial changes in rapidly shifting ones would not be captured in subsequent monitoring cycles. Such an approach has been adopted due to the architecture of the system, in which the *self-optimization* module was verified. In particular, in *self-awareness* all resource utilization metrics are monitored within a single iteration cycle. Consequently, it was not possible to adjust their sampling periods individually. Nevertheless, it should be noted that deploying the *self-optimization* module within different architectures that support adjusting the sampling period for each metric may not require this unification step.

As with the *Shift/Anomaly Detection Model*, the performance of the *Sampling Model* heavily relies on the selection of appropriate parameters as described in the following subsection.

#### 3.4.2. Model Configuration

The parameters used in the AdaM computation are: (1) $T_{min}$, (2) $T_{max}$, (3) λ, (4) γ, with a value between 0 and 1, (5) α, with a value between 0 and 1 and (6) β, with a value between 0 and 1. All of these parameters are described in Table 4. They are configured separately for each sub-model, i.e., the *Resource Sampling Model* and the *Power Consumption Sampling Model*. As the number of parameters is relatively large, relying on their fixed specification, without incorporating domain knowledge, would not be feasible.

Therefore, an approach similar to *Shift/Anomaly Detection Model* was employed. In particular, an external interface that allows the modification of all parameters on the fly was implemented. Through the endpoint *sampling/parameters/type*, a human operator, or another system component, can dynamically adjust the selected model settings. This mechanism works in the same manner as the one introduced for the *Shift/Anomaly Detection Model*, which was described in Section 3.3.2.

It should be easy to notice that the performance of the entire *self-optimization* module relies primarily on its analytical components. Therefore, they have been evaluated in different experimental settings, as described in the following section.

## 4. Experimental Validation

The main objective of the conducted experiments was to evaluate the effectiveness of the implemented algorithms against the selected benchmark datasets and compare them to other state-of-the-art approaches. Here, it should be noted that while frugality was a key design consideration, the current evaluation did not explicitly assess the performance of both models on resource-constrained devices. Yet, such an assessment is recognized as a critical next step and is planned to be included in future research.

As of now, all of the experiments were conducted on a computer with Intel(R) Core(TM) i5-10210U @ 1.60GHz 2.11 GH, 4 Core(s), 16GB RAM. For the assessment, four different real-world datasets were selected:

1. **RainMon monitoring dataset** (https://github.com/mrcaps/rainmon/blob/master/data/README.md, access date: 24 October 2025): an unlabeled collection of real-world CPU utilization data that consists of 800 observations. It was obtained from the publicly available data corpus of the RainMon research project [50]. The CPU utilization traces of this dataset exhibit non-stationary, highly dynamic behavior, including multiple abrupt spikes. Therefore, it provides an excellent basis for validating the effectiveness of adaptive sampling in capturing critical variations in monitoring data.
2. **NAB, synthetic anomaly dataset (*****anomalies jumps-up*****)** (https://github.com/numenta/NAB/blob/master/data/README.md, access date: 24 October 2025): time-series, labeled, dataset composed of 4032 observations, which was obtained from the NAB data corpus [28]. It features artificially generated anomalies that form a periodic pattern. In particular, in this dataset, CPU usage exhibits regular, continuous bursts of high activity, followed by sharp declines in activity. Consequently, it provides a testbed for anomaly detection, allowing the assessment of its contextual anomaly detection capabilities.
3. **NAB, synthetic anomaly dataset (*****load balancer spikes*****)** (https://github.com/numenta/NAB/blob/master/data/README.md, access date: 24 October 2025): similarly to the previous one, a time-series, labeled dataset composed of 4032 observations, which was obtained from the NAB data corpus [28]. It also features artificially generated anomalies, but of different traits than in the *anomalies jumps-up* dataset. In particular, these represent abrupt individual spikes, providing a basis for the evaluation of both the point-based and the contextual anomaly detection.
4. **aerOS cluster IE traces**: resource utilization traces of a single IE that were collected using the *self-awareness* component in the aerOS continuum. They span 87 observations obtained during one hour. Although these traces do not exhibit any substantially abrupt behaviors, they were selected for the analysis since they closely resemble the conditions on which the *self-optimization* algorithms are to operate.

Since the external datasets (i.e., RainMon and NAB) use data formats that differ from the representation of the IE state in the aerOS continuum, their pre-processing was required. In particular, in order to emulate the results retrieved from *self-awareness*, individual scalar values had to be mapped to the resource utilization metrics of the IE. To achieve that, it was necessary to first predefine the test configuration of IE. Accordingly, it was assumed that the IE, which was the subject of monitoring in all conducted tests, had 20 CPU cores, 15615 MB RAM capacity, and 68172 MB disk capacity.

For the RainMon dataset, individual percentage CPU utilization values were converted into a number of utilized CPU cores. A similar operation was conducted for the NAB dataset, since it was assumed that its synthetic traces also represent percentage CPU utilization. In both cases, all remaining monitoring metrics (i.e., RAM and disk utilization) were set to 0 values, since they were not relevant to the experiments.

All of the experiments were executed using a simple, dedicated, test framework that was developed as part of the *self-optimization* module. In particular, the framework emulates the monitoring behavior of *self-awareness* by transforming the continuous time-based process into a discrete-based one. This is achieved by representing fixed temporal intervals (e.g., five seconds) using steps between successive observations in a dataset (e.g., a gap of 5). The instantiated framework allows specifying the configuration of each individual test scenario, which includes: (1) the name of the scenario, (2) a description of the scenario, (3) a specification of IE data, (4) a reference to the JSON file containing monitoring data, (5) list of evaluation metrics, and (6) list of the names and parameters of additional algorithms that are to be used in results comparison. As such, it allows the execution of repeatable experiments for different scenarios. Let us now describe experiments conducted for *Sampling Module* and *Shift/Anomaly Detection Module*.

### 4.1. Sampling Model Verification

The *Sampling Module* was tested on Dataset [data:dataset1]1, Dataset [data:dataset2]2 and Dataset [data:dataset4]4. It was not tested on Dataset [data:dataset3]3, since apart from abrupt metric stream spikes (already present in Dataset [data:dataset1]1), this dataset does not exhibit validation means that would be relevant from the perspective of adaptive sampling.

In all conducted tests, it was assumed that the value of the α parameter and of the β parameter are static, i.e., α=0.5 and β=1. This assumption was made based on the results presented in [34], which indicated that the α and β do not have a significant impact on the performance of the model. Similarly, the minimal $T_{min}$ and the maximal $T_{max}$ sampling periods were fixed to the values determined after consultation with the creators of the *self-awareness* module, i.e., $T_{min}=1000$ (ms) and $T_{max}=6000$ (ms). This ensured that the chosen values closely reflect the real-world conditions.

The only parameters that were not statically defined were multiplicity λ and imprecision γ. Therefore, in order to select their best combination, comparative tests were conducted on each dataset. First, the focus was to establish the best γ. Thus, λ was fixed to 1000, which is a reasonable baseline as it represents a multiplicity of 1 (no multiplicity). After selecting the most optimal γ, it was used in tests that have been aiming at selecting the best λ.

The assessment was based on two different quality measures. First (following [34]) was the Mean Absolute Percentage Error (MAPE). It allows illustrating the error between the predicted stream values and the actual ones. The formula for MAPE is illustrated in Equation (14), where $x_{actual}$ is an actual value, $x_{predicted}$ is a predicted value (set to the last monitored value, if the observation is skipped), and *n* is the size of the sample.

(14) $$MAPE=\frac{\sum_{i}^{n}\frac{|x_{predicted}-x_{actual}|}{x_{actual}}}{n}\cdot 100$$

A second metric was the Sample Ratio (SR), which quantifies the percentage of samples that were monitored. This measure provides insight into the reduction in the data dissemination volume. It is computed using Equation (15).

(15) $$SR=\frac{n_{monitored}}{n_{actual}}\cdot 100$$

Those two quality metrics were used to compute the Joint-Performance Metric (JPM), which is formulated as in Equation (16). Given that both MAPE and SR are to be minimized, in order to achieve the best performance, JPM serves as an aggregate that evaluates the overall trade-off between these goals. Here, the higher JPM indicates better effectiveness.

(16) $$JPM=100-\frac{MAPE+SR}{2}$$

Table 5 presents the results of the tests executed for different γ values for all three datasets. In the case of the aerOS dataset, the MAPE was calculated by averaging the MAPE of all metrics (i.e., CPU, RAM, and disk utilization).

As can be observed, the best results were achieved for γ=0.6. In the majority of cases, SR exhibited a decreasing trend as γ increased, which is consistent with the expected behavior. In particular, higher tolerance of estimation errors expressed through γ leads to longer sampling intervals, which, as a consequence, reduces the number of monitored samples. Interestingly, for γ=1.0, the SR unexpectedly increased in all conducted tests, achieving slightly degraded overall performance (JPM). Additionally, the experiments conducted on the aerOS dataset yielded the lowest SR values (between 80 and 100%) compared to the NAB and RainMon. This difference may potentially be attributed to the fact that the sampling period computation on the aerOS dataset was performed on multivariate observations (i.e., it accounted for RAM and disk utilization), making the final sampling frequency dependent on the distribution of all three utilization metrics.

**Figure 4:**
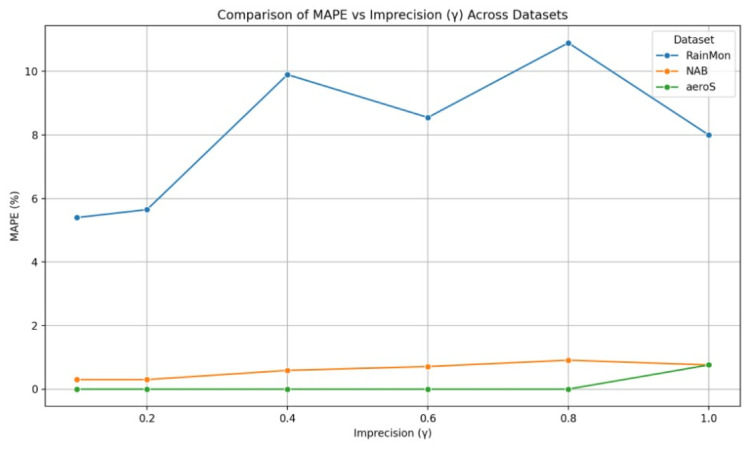
The comparison of achieved MAPE values for different imprecisions and datasets.

Moreover, it is observable that the MAPE values are significantly below the acceptable imprecision γ in all cases. However, the results of tests conducted on different datasets exhibit significant differences in the behavior of this metric, as illustrated in Figure 4.

For the RainMon dataset, the MAPE values were fairly regular and oscillated between 5 and 10%. On the other hand, for the NAB dataset, the MAPE values were the lowest, with almost all of them being below 1%. Finally, for the aerOS dataset, the average MAPE values were the least regular, making it difficult to observe any trends. These outcomes emphasize the criticality of adjusting the model parameters for the different application scenarios.

Based on the observed results, the value of γ was fixed at 0.6. Next, it was used in the selection of the multiplicity parameter (λ). For λ, four different values were considered: 500, 1000, 2000, and 3000. Table 6 summarizes the outcomes of the tests.

The obtained results demonstrate that λ and MAPE exhibit a positive correlation, while λ and SR have an inverse relationship. Since the changes in MAPE are relatively small (being significantly below the considered imprecision parameter), a clear increase also appears in the case of JPM. Therefore, in the non-critical applications, where a moderate level of estimation error is tolerable, selecting a higher value of λ may be beneficial. However, in cases where precision is critical, the overall JPM value might be of secondary importance to maintaining a lower MAPE.

Taking into account the aforementioned findings, the combination of γ=0.6 and λ=3000 was considered to be the most optimal. As such, it was used in the subsequent experiments evaluating the performance of the implemented adaptive sampling approach against other existing algorithms. In particular, the (1) UDASA [30] and (2) AWBS [29] adaptive sampling techniques were used in the assessment. However, it should be stressed again that establishing these values has to be performed for each application of the proposed approach.

For UDASA, the following parameters were applied: (1) window size N=15, (2) saving level (controls the level of precision) n=5, and (3) baseline sampling period T=1000. These parameters were determined by analyzing the results of the experiments presented in [29] and adjusting them to the specificity of test datasets (e.g., window size decreased due to a smaller number of observations).

Similarly, for the AWBS, the parameters included: (1) maximal window size mx=6, (2) initial window size $w_{initial}=1$, and (3) threshold (used to determine anomalies) th=40%. Here, let us note that in the original version of AWBS, the threshold was not parametrized but determined with respect to the percentage difference of the first two moving averages. However, this approach does not capture edge cases (e.g., when the percentage difference is 0), which appear in the considered test datasets. In such scenarios, since the threshold is static, it does not adapt to the evolution of a metric stream (i.e., it would remain 0). Consequently, there is almost no sampling adjustment. To mitigate that, it was decided that the threshold should be predefined. Hence, it was set to 40% so that it corresponds to the imprecision parameter γ. The results of all experiments are summarized in Table 7.

**Figure 5:**
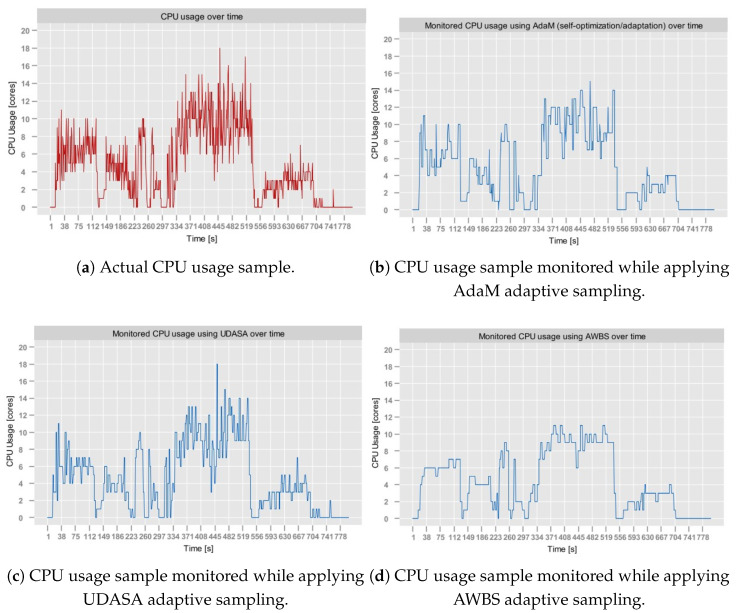
Results of adaptive sampling on the RainMon dataset.

It can be observed that in terms of JPM, the AdaM algorithm implemented in *Sampling Model* yields comparable results to the remaining state-of-the-art approaches. It outperformed UDASA in all executed tests while achieving slightly worse outcomes (by around 2%) than AWBS. Among the evaluated algorithms, UDASA demonstrated the least effective data volume reduction, which was associated with a relatively high prediction error. This is clearly observable in Figure 5, which depicts the monitoring of the RainMon dataset under various adaptive sampling strategies. While UDASA is able to account for spikes with a larger magnitude, it smooths out smaller fluctuations, which contributes to the prediction error.

**Figure 6:**
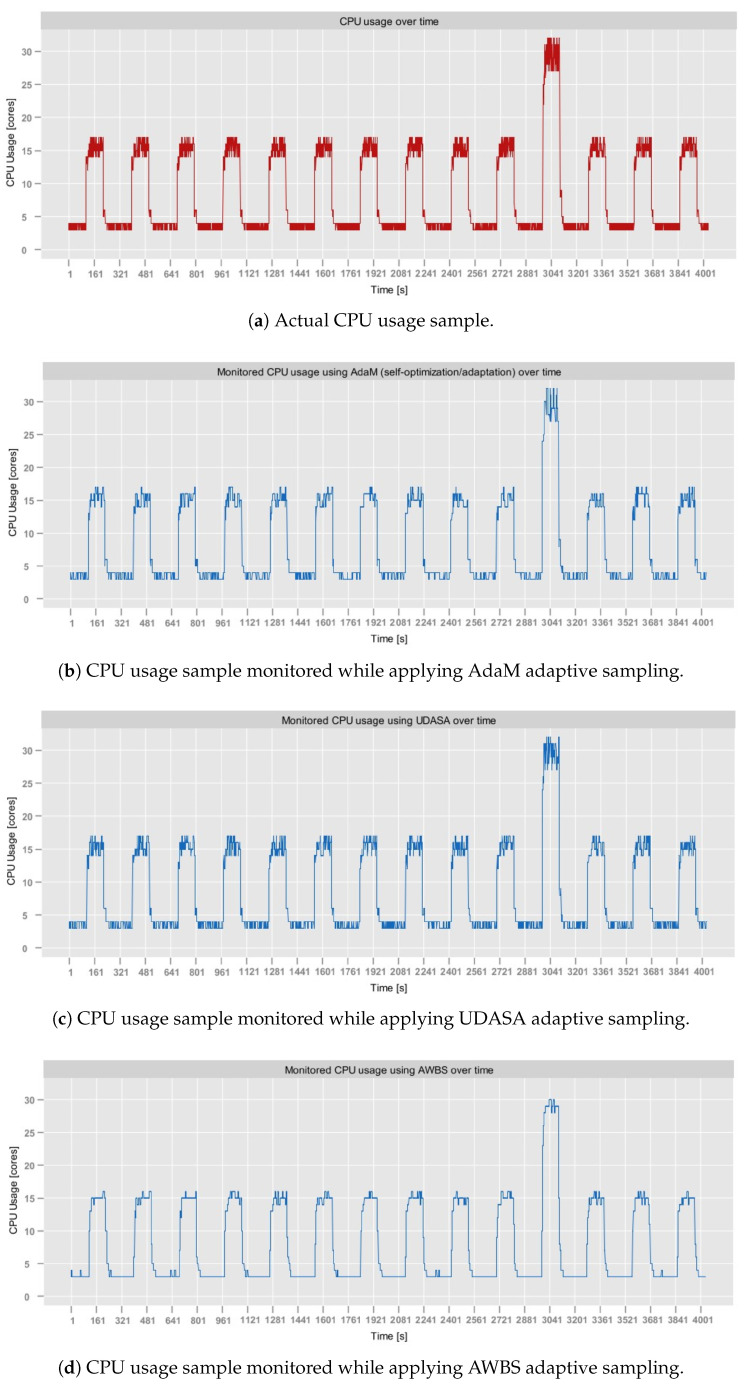
Results of adaptive sampling on NAB (*anomaly jumps-up*) dataset.

On the contrary, in terms of the data volume reduction (SR), the best performance, among all algorithms, was achieved by AWBS. However, it should be noted that this result was accompanied by significantly higher prediction error, as indicated by substantially worse MAPE values. This is reflected, among others, in Figure 6, where the results of adaptive data sampling on the NAB dataset are demonstrated. In AWBS, large portions of fluctuations are bypassed by averaging them within sliding windows. As a result, despite the fact that it significantly reduces the amount of disseminated data, it accounts for substantial delays in capturing the evolution of the metric stream, which manifests in the estimation errors.

**Figure 7:**
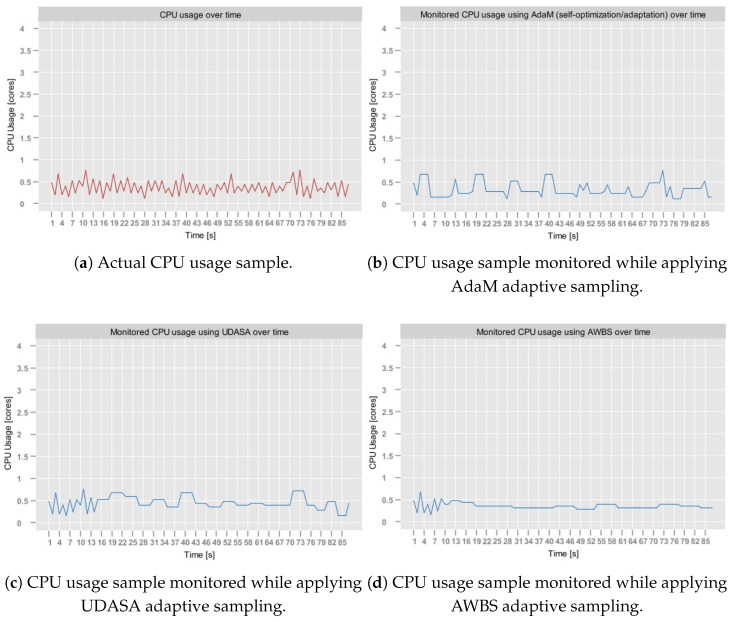
Results of adaptive sampling on aerOS dataset.

Overall, it can be concluded that among all presented algorithms, the implemented AdaM approach offers the most balanced trade-off between quality and performance. This observation is supported by the results of the tests carried out on the aerOS dataset, which are presented in Figure 7. Here, the CPU utilization exhibited highly systematic fluctuations with relatively low amplitude. Moreover, there were only a few observations that did not follow this pattern.

It is also noticeable that AWBS smoothed the majority of observations, failing to capture any of the isolated spikes. The only apparent fluctuations that it detected appeared at the beginning of the metric stream. This is likely due to an insufficient number of data samples to compute the average. In comparison, UDASA performed slightly better, as it was able to capture some of the variations. However, it failed to immediately account for the spikes, which are visible in Figure 7c by shifted slopes. Additionally, UDASA rarely adjusted to decreasing values. Finally, the AdaM technique had greater responsiveness to more substantial fluctuations, even though they were not of a large magnitude.

It can be concluded that among the evaluated frugal adaptive sampling algorithms, AdaM is able to best accommodate different types of metric streams, including not only those characterized by abrupt changes but also those with a stable behavior. The experiments that were conducted proved that the method is able to respond quickly to substantial fluctuations, adjusting the sampling frequency accordingly. Furthermore, its performance aligns well with the operational characteristics of real-life ECC, as demonstrated in the tests executed over monitoring data obtained from the aerOS continuum.

Let us now describe the evaluation of the algorithm implemented in *Shift/Anomaly Detection Model*.

### 4.2. Shift/Anomaly Detection Model Verification

The process of evaluating the *Shift/Anomaly Detection Model* can be divided into two main parts: (1) assessing the quality of the anomaly detection using labeled datasets and comparing its performance to other existing state-of-the-art approaches, and (2) running the *Shift/Anomaly Detection Model* on unlabeled aerOS data, and consulting the obtained results with aerOS domain-experts to assess their accuracy.

For the first part, Dataset [data:dataset2]2 and Dataset [data:dataset3]3 were used. Both of them provide a ground-truth mask, which, for each anomaly, specifies a time interval during which it is supposed to be detected. In this context, Dataset [data:dataset1]1 was not selected for the tests, since it does not provide any labels enabling the distinguishing of the anomaly observations from the normal ones.

In all conducted experiments, it was assumed that the main objective was to detect anomalies as soon as possible. Hence, the parameters $W_{normal}$ and $W_{anomaly}$ were fixed and set to the value 1. In this way, the model was able to respond to abrupt fluctuations without delaying alerts regarding anomalous observations. Similarly, the value of $Th_{normal}$ was fixed to 0.05, since the objective was to investigate the performance of the transition between the normal and the anomalous state, and not the other way around. Consequently, the only parameter that had to be adjusted was $Th_{anomaly}$. Therefore, in order to establish its optimal value, a set of comparative experiments was conducted.

The performance of the anomaly detection was numerically assessed using the normalized anomaly score (S) quality measure. This measure was introduced as part of NAB [28], and it weights individual detections, relative to the expected time interval (anomaly window) in which they were supposed to be captured. In particular, it works in the following way. Detected anomalies are classified into three categories: (1) detected before the anomaly window, (2) detected within the anomaly window, and (3) detected after the anomaly window. Each such detected anomaly is associated with its individual score, which indicates how much a given detection should contribute to the overall quality of the algorithm. The detection score of individual anomalies is computed using a sigmoid function according to Equation (17).

(17) $$\sigma \left(y_{i}\right)=\left\{\begin{array}{cc} -1, & \mathrm{if}y_{i}\mathrm{before}\mathrm{window}\\ 2\cdot \frac{1}{1+e^{5y}}-1, & \mathrm{otherwise} \end{array}\right.$$

In this equation, $y_{i}$ represents the position of the detected anomaly *i*, relative to the anomaly window. It is calculated using the formula in Equation (18).

(18) $$y_{i}=\left\{\begin{array}{cc} \frac{idx_{anomaly}-idx_{start\_window}}{window\_size}, & \mathrm{if}y_{i}\mathrm{inside}\mathrm{window}\\ \frac{idx_{end\_window}-idx_{anomaly}}{windw\_size}, & \mathrm{otherwise} \end{array}\right.$$

As can be observed, whenever the anomaly detection ($idx_{anomaly}$) is closer to the beginning of the anomaly window ($idx_{start\_window}$), the relative position goes to −1, hence, resulting in a larger positive value of the detection score. Therefore, early detection of anomalies is prioritized. On the other hand, when the anomaly is detected just at the end of the anomaly window ($idx_{end\_window}$), then the relative position is 0, neutralizing the impact of its detection score. For the anomalies that go beyond the window, the value of the detection score becomes increasingly negative as their relative distance from the detection window grows. Readers interested in understanding a more in-depth rationale behind applying such formulas are encouraged to read [28].

After scoring individual detections, they are weighted (see Equation (19)) using true positive weight ($W_{tp}$), and false positive weight ($W_{fp}$). Then, all weighted individual detections are summed. Lastly, the number of windows with undetected anomalies ($n_{undetected}$), weighted based on the false negative weight ($W_{fn}$), is subtracted from the sum. Hence, the overall formula of a score is as presented in Equation (20).

(19) $$\sigma^{A}\left(y_{i}\right)=\left\{\begin{array}{cc} \sigma \left(y_{i}\right)\cdot W_{tp}, & \mathrm{if}y_{i}\mathrm{inside}\mathrm{window}\\ \sigma \left(y_{i}\right)\cdot W_{fp}, & \mathrm{otherwise} \end{array}\right.$$

(20) $$S_{n}=\sum_{i}^{n}\sigma^{A}\left(y_{i}\right)-n_{undetected}\cdot W_{fn}$$

In the experiments, the normalized formula of the score was used as outlined in Equation (21).

(21) $$S=100\cdot \frac{S_{n}-S_{null}}{S_{perfect}-S_{null}}$$

Here, (1) $S_{perfect}$ represents a perfect score (i.e., all anomalies recognized at the beginning of their anomaly windows), and (2) $S_{null}$ represents a case when no anomalies were detected (i.e., baseline defined individually for each NAB dataset).

The *S* was utilized as a basis for assessing the quality of the anomaly detection.

It should be noted that the standard weighting profile, proposed by the authors of NAB, makes the anomaly score extremely sensitive to false positives. Specifically, for standard value $W_{fp}=0.11$, the anomaly score $S_{n}$ attains large negative values in scenarios characterized by a large number of false positives (e.g., when the model detects point anomalies, rather than contextual ones). At the same time, the scale of the possible false positives is not accounted for in the $S_{null}$ computation. As a result, under such conditions, the overall normalized $S_{n}$ value may fall below 0, violating the intended 0–100% range. In order to accommodate this issue, in the conducted experiments, the standard weighting profile was modified by lowering the $W_{fp}$ to 0.05.

In this context, the results of tests conducted on all datasets using different values of $Th_{anomaly}$ are presented in Table 8.

The results illustrate that for low threshold values (i.e., $Th_{anomaly}=0.1$), the model fails to detect any anomalies, hence the score *S* is 0%. Then, for slightly larger $Th_{anomaly}$ (i.e., $Th_{anomaly}=0.2$ or $Th_{anomaly}=0.3$), *S* begins to sharply increase. It can be attributed to the fact that for such parameters, the system remains moderately sensitive, rejecting minor fluctuations (i.e., minimizing false positives) while detecting substantial spikes. The peak results are obtained for $Th_{anomaly}=0.3$ (93.12%) in the case of *anomaly jumps-up* and $Th_{anomaly}=0.2$ (21.47%) for *load balancer spikes*. After reaching this value, the performance begins to steadily decline. It can be a result of an increased sensitivity of the system, hence a larger number of false positive anomalies. Moreover, it should be noted that the NAB datasets label the contextual anomalies. Consequently, although the density-based anomaly detection algorithm implemented in *Shift/Anomaly Detection Model* is able to detect the point-based anomalies, it may not be well suited for the contextual ones.

**Figure 8:**
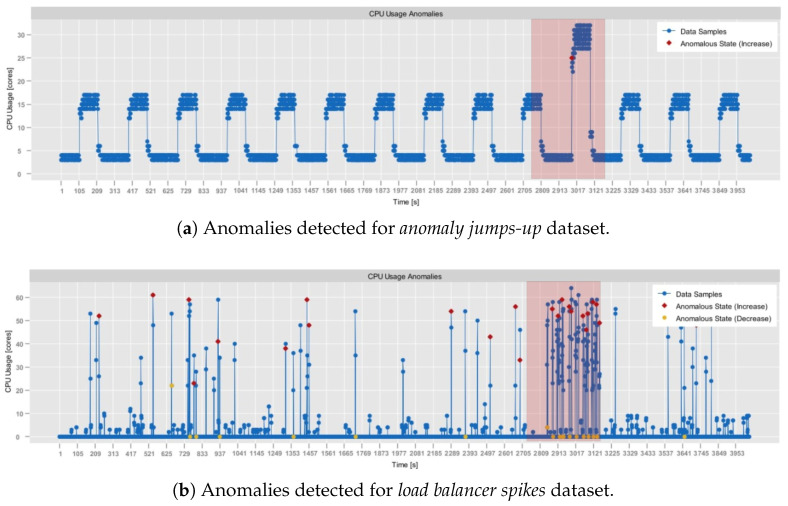
Results of anomaly detection for $Th_{anomaly}=0.3$ and NAB datasets (*anomaly jumps-up* and *load balancer spikes*).

This last conclusion is particularly supported by the *S* results obtained for the *load balancer spikes* dataset, which are significantly lower than in the case of *anomaly jumps-up*. It can be observed in Figure 8, which presents the comparison of the anomalies detected for *anomaly jumps-up* (Figure 8a) and *load balancer spikes* (Figure 8b) for $Th_{anomaly}=0.3$. In the figures, the red semi-transparent area represents the labeled anomaly window. In the case of *anomaly jumps-up*, only one anomaly was detected because there was only one fluctuation of large magnitude. However, in the case of *load balancer spikes*, the data stream had multiple high spikes, each of which was classified as an anomaly, even though they were outside of the anomaly window. It is clearly visible that the algorithm was not able to recognize the anomalies based on the periodicity and frequency of their occurrences.

Based on the obtained results, the threshold $Th_{anomaly}=0.3$ was selected to be used in subsequent experiments, comparing the performance of *Shift/Anomaly Detection Model* to other existing state-of-the-art approaches. In particular, for the comparison, the Contextual Anomaly Detector CAD-OSE [51] and ARTime [52] were used. Both these algorithms were implemented as part of NAB and achieved the best results on the NAB scoreboard when tested against different corpus datasets. Therefore, it was decided that they would serve as a suitable basis for the performance evaluation. The obtained results, for running *Shift/Anomaly Detection Model*, CAD-OSE and ARTime on *anomaly jumps-up* and *load balancer spikes* are summarized in Table 9.

**Figure 9:**
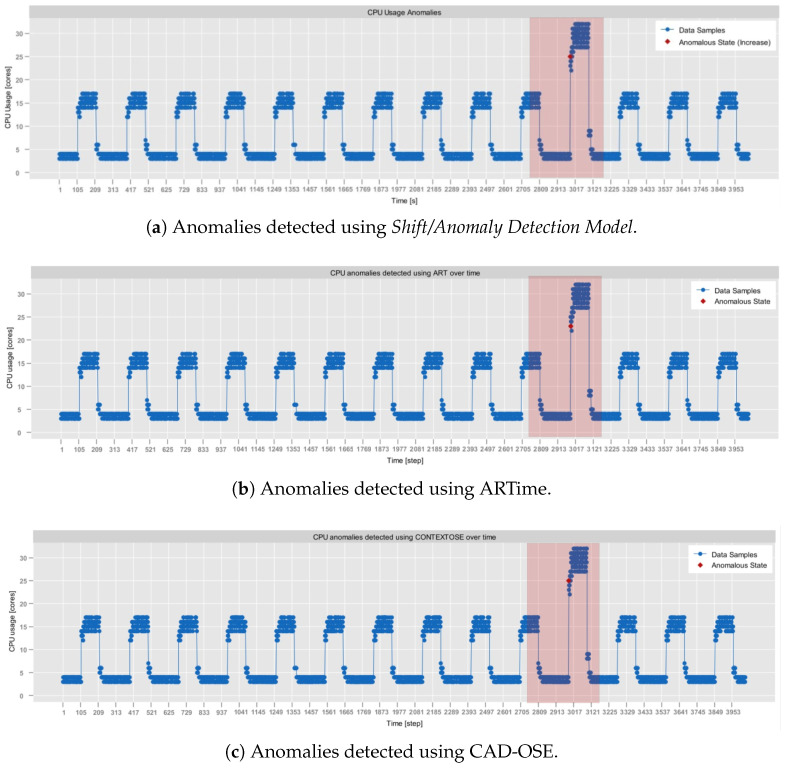
Results of anomaly detection on NAB (*anomaly jumps-up*).

**Figure 10:**
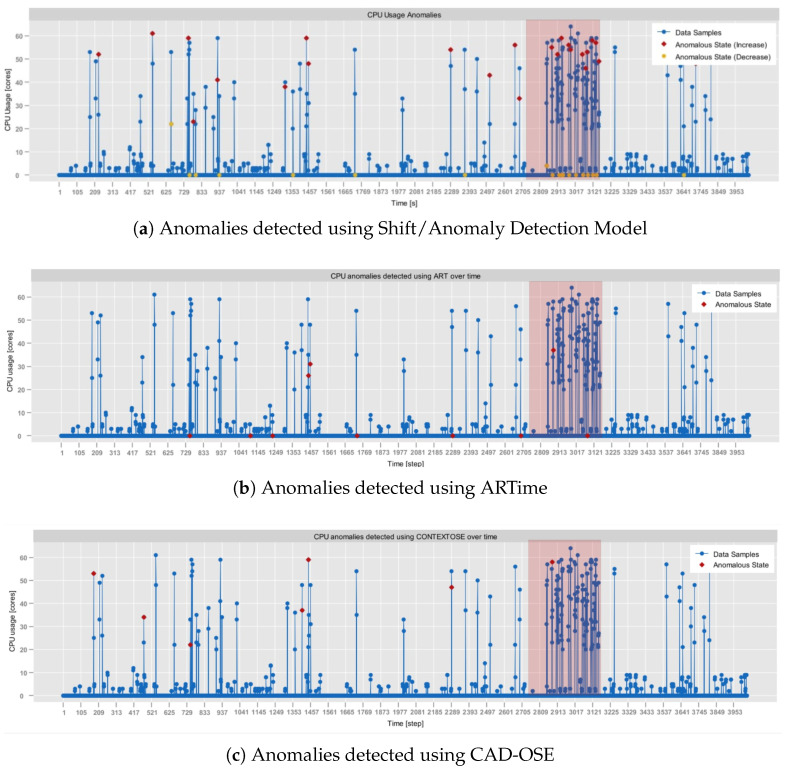
Results of anomaly detection on NAB (*load balancer spikes*) dataset.

As can be observed, the density-based algorithm implemented in *Shift/Anomaly Detection Model* achieved a comparable performance to the remaining tested approaches. In particular, for both datasets, it scored higher, or equal, to CAD-OSE, but slightly lower than ARTime. The superior performance achieved in both scenarios by ARTime can be primarily attributed to its inherent complexity, as it is based on the Adaptive Resonance Theory (ART) neural network. Consequently, among all applied algorithms, it most effectively captures contextual anomalies and reduces false positive rates. Moreover, since ART networks are designed to quickly adapt to new input patterns without retraining, ARTime is able to respond immediately to the detected deviations. It is clearly illustrated in Figure 9 and Figure 10 that depict anomaly detection results in both the *anomaly jumps-up* and the *load balancer spikes* datasets.

In the case of *anomaly jumps-up* dataset (Figure 9), all three approaches were able to capture the anomaly within the intended anomaly window. Furthermore, none of them detected false positives. Here, the difference in their performance, reflected by the value of *S*, arises from the time at which the anomalies were detected. Specifically, ARTime was the fastest in anomaly detection, followed by the *Shift/Anomaly Detection Model*, with CAD-OSE demonstrating the slowest response. However, in this particular case, the differences in anomaly detection time were negligible. Therefore, in applications where rapid anomaly detection is not highly critical (e.g., monitoring trends in streaming sensor data), all three algorithms can be considered to be equivalent in terms of performance.

On the other hand, for the experiments performed on the *load balancer spikes* dataset (Figure 10), the differences are more prominent. In particular, all algorithms detected a substantial number of false positive observations, where their highest number was detected by *Shift/Anomaly Detection Model*. This strengthens the conclusion that the implemented density-based algorithm is better suited for point anomaly detection rather than contextual anomalies. Nevertheless, it is important to note that the *Shift/Anomaly Detection Model* detected the true anomaly relatively early, within the intended anomaly window, which contributed positively to its overall anomaly score. In contrast, the number of false positives detected by ARTime and CAD-OSE was comparably lower. The variation in the final performance, among these two algorithms, resulted from the time in detecting the true anomaly, with ARTime identifying it significantly faster than CAD-OSE.

The conducted experiments showcase that ARTime achieves the best overall performance. However, it is important to emphasize that this particular algorithm is very computationally complex, which hinders its applicability in environments that perform operations on resource-restricted devices (e.g., edge sensors). Consequently, due to the comparable performance achieved by the *Shift/Anomaly Detection Model*, it may be an appropriate replacement for the anomaly detection in environments that require frugality of computational resources.

**Figure 11:**
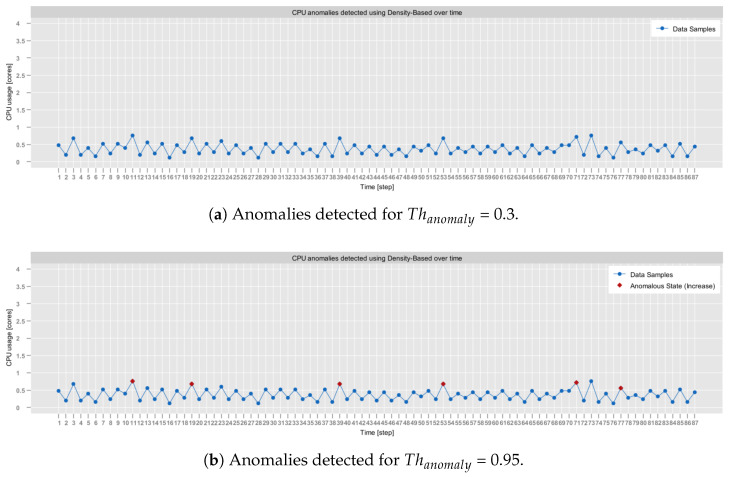
Results of anomaly detection on aerOS dataset.

In order to assess the performance of *Shift/Anomaly Detection Model* in close to the real-life conditions, the final tests were conducted on the Dataset [data:dataset1]1 (i.e., the aerOS dataset). Since the aerOS dataset is characterized by low data fluctuations, the experiments were conducted first for $Th_{anomaly}$ = 0.3 and then for $Th_{anomaly}$ = 0.95. The main purpose was to evaluate the behavior of the model under increased sensitivity to anomaly detection. The results obtained for both configurations are illustrated in Figure 11. Here, it should be noted that the aerOS dataset does not provide ground-truth labels, describing which observations are anomalous. Therefore, the reliability of the obtained results has to be confirmed by the aerOS experts.

As can be observed, for the $Th_{anomaly}$ = 0.3, the *Shift/Anomaly Detection Model* detected no anomalies. This behavior is fully rational, as in such a configuration, the model will not be sensitive to minor fluctuations, such as those observed in the aerOS dataset. Singular anomalies were detected only after increasing $Th_{anomaly}$ to a value of 0.95, representing a very high sensitivity setting. Under this configuration, the model identified several small spikes. Although in this particular case, they illustrate mainly that the sensitivity of the *Shift/Anomaly Detection Model* can be effectively and seamlessly tuned via its parameters.

The results of the experiments demonstrate the model’s versatility, making it adaptable for a wide range of application domains, ranging from those that require the detection of subtle variations (e.g., healthcare applications) to those that should respond only to more substantial fluctuations (e.g., industrial fault detection).

## 5. Limitations

The implemented approach was evaluated on a set of synthetic and real-world datasets, proving its effectiveness by showing that individual models achieve performance comparable to or better than other existing state-of-the-art approaches. However, there are still significant limitations that need to be addressed further.

For instance, the implemented *Shift/Anomaly Detection Model* is able to perform well only on the point-based anomalies, hindering its applicability in systems that rely on long-term contextual information (e.g., identifying anomalies in network traffic). Hence, future research should explore possibilities of incorporating context into the anomaly detection process, while still preserving the frugal nature of the approach.

Separately, the *Sampling Model* has been primarily tested on the univariate metric streams, under-representing the real-world applications that rely on multivariate data. As such, a broader validation within the multivariate data domain is required to fully assess the performance of the model.

Moreover, as demonstrated by the results of the experiments, both models are highly reliant on their parameters, requiring substantial knowledge from domain experts for their tuning. Although this has been addressed partially by introducing interfaces supporting on-the-fly parameters’ modification, the current solution is fully manual and may not be feasible in large-scale complex systems. Consequently, future efforts should investigate automated mechanisms for parameter tuning or potentially propose means for autonomous *self-configuration*.

Finally, while the current study validates the proposed *self-optimization* module in simulation and dataset-driven scenarios, its real-world efficacy remains to be assessed. This limitation is particularly relevant since only experiments conducted on real-life hardware can capture the true impact of the algorithms from the point of view of actual resource utilization and energy consumption. Furthermore, since frugality was a central aspect of the design of the presented algorithms, so that they can accommodate resource-restrictive devices, experiments performed solely in simulated environments cannot fully verify whether this objective was achieved. Therefore, future deployments and experiments of the *self-optimization* module must be conducted on physical ECC infrastructures. Such evaluations would require the careful selection and configuration of edge devices, as well as the design of representative test scenarios along with domain-specific evaluation metrics. Given the extensive scope of these experiments, they fall beyond the limits of the present contribution but will be undertaken as a continuation of the current research.

Nevertheless, the preliminary results obtained within this contribution indicate a strong potential in the implemented *self-optimization* module, driven by its modularity, generalizability, and frugal approach.

## 6. Concluding Remarks

The contribution introduced a novel perspective on the *self-optimization* for resource-constrained edge devices that transmit and process streaming data. Specifically, it presented a design of a modular and pluggable *self-optimization* module comprising two core components: (1) *Shift/Anomaly Detection Model* and (2) *Sampling Model*. In light of an ambiguity existing in the current literature regarding the definition of *self-optimization*, it can be stated that, in the context of this work, *self-optimization* was perceived from the perspective of enhancing operational efficiency and minimizing energy consumption.

On the one hand, in order to reduce the energy overhead associated with the transmission of large data volumes, the focus was placed on the optimization of the data-monitoring process. The PEWMA-based adaptive sampling technique, implemented within *Sampling Model*, was utilized to autonomously adjust the monitoring rate, reducing the overall disseminated data volume while preserving the relevant information. Such an approach bridges a significant gap in the existing *self-optimization* literature, where the subject of optimizing the data transmission process is often overlooked.

On the other hand, through *Shift/Anomaly Detection Model*, this study also addressed the challenges of resource optimization by analyzing the utilization of current resources and detecting potential abnormalities. However, it should be noted that this process pertained only to the detection of possible adaptation possibilities, rather than suggesting particular actions to undertake. By doing so, the presented approach provides a greater scope for generalization since the adaptation actions are often highly context-dependent and application-specific. Following such a design enables the *self-optimization* module to be seamlessly integrated into different infrastructures, where it can interface with external components responsible for executing adaptations. Nevertheless, the examples of such integrations are yet to be studied in future publications.

Moreover, it should be noted that the algorithms implemented in both models were derived based on the analysis of the current state-of-the-art, where the frugality, understood from (1) the resource utilization, and (2) required data volume perspectives, constituted a primary criterion behind the algorithm’s selection. Consequently, it can be argued that the introduced approach accommodates well the requirements of a wide variety of ECC applications, including those with substantial processing limitations. For instance, the developed *self-optimization* module may be equally deployed on ECC composed of large edge servers processing satellite images, but also on embedded devices located within vehicles, or small wearable smartwatches used to monitor vital parameters of patients.

As such, it can be summarized that the implemented *self-optimization* module may constitute a viable component for next-generation intelligent ECC.

## Figures and Tables

**Table 1:** Summary of different adaptive sampling algorithms.

Algorithm	Adaptation Target	Method	Batch- Based	General Purpose	Extra Storage
*AWBS* [29]	window size	moving average	X	X	
*ApproxECIoT* [35]	window size	standard deviation	X		
*UDASA* [30]	sampling period	MAD	X	X	
*AdaM* [34]	sampling period	PEWMA		X	
*Linear Regression* [36]	sampling period	linear regression	X	X	X
*Kalman Sampling* [37]	sampling period	Kalman filter	X	X	X

**Table 2:** Summary of different anomaly detection algorithms.

Algorithm	Mode	Anomaly Type	Method	Concept Drift	Extra Storage
*LightESD* [39]	semi-online	contextual	periodograms	X	X
*APD-HT/CC* [40]	offline	contextual	k-means		X
*CAD* [41]	semi-online	contextual	LSTM	X	X
*PiForest* [42]	online	point/contextual	decision tree	X	X
*Density-based* [44]	online	point	data density	X	
*EFDT* [45]	online	point	decision tree		X

**Table 3:** Notation used in the density-based anomaly detection algorithm.

Symbol	Description
$x_{i}$	Observed value at time *i*.
*N*	Size of the sample.
*K*	Number of consecutive observations of the same state (i.e., anomalous or normal).
$\mu_{i}$	Sample mean at the time *i*.
$\sum_{i}$	Sample scalar product at the time *i*.
$D_{i}$	Density at the time *i*.
${\bar{D}}_{i}$	Average density at the time *i*.
$\Delta D_{i}$	Difference between consecutive densities at the time *i*.
$Th_{anomaly}$	Threshold/tolerance weight put on the average density during the transition to the anomalous state.
$Th_{normal}$	Threshold/tolerance weight put on the average density during the transition to the normal state.
$W_{anomaly}$	Window of consecutive observations that must be determined as anomalies in order to switch to the anomalous state.
$W_{normal}$	Window of consecutive observations that must be determined as normal in order to switch to the normal state.

**Table 4:** Notation used in the AdaM adaptive sampling algorithm.

Symbol	Description
$x_{i}$	Observed value at time *i*.
*N*	Size of the sample.
$\delta_{i}$	Distance between two consecutive observation at time *i*.
$\mu_{i}$	PEWMA representing the estimated metric stream evolution at time *i*.
$\nu_{i}$	PEWMA of the squared values (second-order statistic) at time *i*.
$\sigma_{i}$	Standard deviation at time *i*.
${\bar{\sigma }}_{i}$	Moving standard deviation at time *i*.
$c_{i}$	Confidence interval that measures the accuracy of metric evolution estimation at time *i*.
$P_{i}$	Probability of $\delta_{i}$ at time *i*.
$T_{i}$	Sampling period estimated at time *i*.
$T_{min}$	Minimal sampling period value.
$T_{max}$	Maximal sampling period value.
λ	Optimal multiplicity that scales the estimated sampling period value.
γ	Imprecision used to calculate the acceptable confidence of the estimation.
α	Weighting factor put on the value in PEWMA calculation.
β	Weighting factor put on the probability in PEWMA calculation.

**Table 5:** Results of the tests of different γ values obtained for all three datasets.

Results for RainMon Dataset.			
γ	MAPE	SR	JPM
0.1	5.40%	56.62%	68.98%
0.2	5.65%	47.87%	73.24%
0.3	9.15%	49.87%	70.48%
0.4	9.9%	40.12%	74.99%
0.5	9.91%	37.12%	76.48%
0.6	8.55%	37.12%	**77.16%**
0.7	11.92%	34.62%	76.72%
0.8	10.9%	37%	76.05%
0.9	10.92%	36.75%	76.16%
1.0	8%	45%	73.5%
**Results for NAB (** ***Anomaly Jumps-Up*** **) Dataset.**			
** γ **	**MAPE**	**SR**	**JPM**
0.1	0.42%	49.45%	75.06%
0.2	0.3%	43.32%	78.18%
0.3	0.34%	38.29%	80.67%
0.4	0.59%	33.11%	83.14%
0.5	1.11%	28.62%	85.13%
0.6	0.71%	27.38%	85.94%
0.7	0.24%	27.03%	**86.35%**
0.8	0.91%	27.13%	85.97%
0.9	0.44%	27.33%	86.11%
1.0	0.76%	30.05%	84.58%
**Results for aerOS Dataset.**			
** γ **	**Δ MAPE**	**SR**	**Δ JPM**
0.1	0%	100%	50%
0.2	0%	98.85%	50.57%
0.3	0.38%	93.10%	53.25%
0.4	1.14%	86.20%	56.31%
0.5	3.06%	81.60%	57.65%
0.6	4.98%	60.91%	**66.54%**
0.7	0.38%	95.40%	51.93%
0.8	0%	86.20%	50.57%
0.9	0%	87.35%	56.32%
1.0	0.76%	87.35%	55.94%

*Note:* In the table, the best outcomes are highlighted using bold formatting.

**Table 6:** Results of the tests of different λ values for all three datasets.

Results for RainMon Dataset.			
λ	MAPE	SR	JPM
500	5.27%	51.37%	71.67%
1000	8.55%	37.12%	77.16%
2000	14.92%	29.75%	77.66%
3000	14.28%	24.37%	**80.67%**
**Results for NAB (** ***Anomaly Jumps-Up*** **) Dataset.**			
** λ **	**MAPE**	**SR**	**JPM**
500	0.64%	36.48%	81.43%
1000	0.71%	27.38%	85.94%
2000	0.94%	23.31%	87.87%
3000	0.74%	21.97%	**88.64%**
**Results for aerOS Dataset.**			
** λ **	**Δ MAPE**	**SR**	**Δ JPM**
500	0%	91.95%	54.022%
1000	4.98%	60.91%	66.54%
2000	1.91%	75.86%	61.11%
3000	2.68%	41.37%	**77.96%**

*Note:* In the table, the best outcomes are highlighted using bold formatting.

**Table 7:** Comparison of the performance of all selected algorithms run against all three datasets.

Results for RainMon Dataset.			
Algorithm	MAPE	SR	JPM
AdaM (*self-optimization*)	14.28%	24.37%	80.67%
UDASA	9.41%	37%	76.79%
AWBS	14.46%	19%	**83.26%**
**Results for NAB (** ***Anomaly Jumps-Up*** **) Dataset.**			
**Algorithm**	**MAPE**	**SR**	**JPM**
AdaM (*self-optimization*)	0.74%	21.97%	88.64%
UDASA	0.79%	35.19%	82.0%
AWBS	1.53%	16.96%	**90.74%**
**Results for aerOS Dataset.**			
**Algorithm**	**Δ MAPE**	**SR**	**Δ JPM**
AdaM (*self-optimization*)	2.68%	41.37%	77.96%
UDASA	10.34%	39.08%	75.28%
AWBS	8.74%	30.1%	**80.58%**

*Note:* In the table, the best outcomes are highlighted using bold formatting.

**Table 8:** Results of the tests of different $Th_{anomaly}$ values obtained for both NAB datasets.

Results for NAB (*Anomaly Jumps-Up*) Dataset.	
${\mathrm{Th}}_{\mathrm{anomaly}}$	S
0.1	0%
0.2	0%
0.3	**93.12%**
0.4	90.61%
0.5	87.57%
0.6	65.43%
0.7	19.15%
0.8	9.99%
0.9	3.12%
**Results for NAB (** ***Load Balancer Spikes*** **) Dataset.**	
${\mathrm{Th}}_{\mathrm{anomaly}}$	**S**
0.1	0%
0.2	**73.48%**
0.3	72.42%
0.4	59.89%
0.5	44.96%
0.6	27.32%
0.7	6.73%
0.8	1.24%
0.9	0.44%

*Note:* In the table, the best outcomes are highlighted using bold formatting.

**Table 9:** Comparison of the performance of different anomaly detection algorithms run against NAB *anomaly jumps-up* and *load balancer spikes* datasets.

Results for *Anomaly Jumps-Up* Dataset.	
Algorithm	S
Density-based (*self-optimization*)	93.12%
CAD-OSE	93.10%
ARTime	**100%**
**Results for NAB (** ***Load Balancer Spikes*** **) Dataset.**	
**Algorithm**	**S**
Density-based (*self-optimization*)	62.42%
CAD-OSE	52.82%
ARTime	**77.10%**

*Note:* In the table, the best outcomes are highlighted using bold formatting.

## Data Availability

NAB datasets used as part of this study can be found at: https://github.com/numenta/NAB (Access date: 24 October 2025). The aerOS dataset presented in this article is not readily available yet because it is a subject of an ongoing study as part of the aerOS project and will be published along with the completed open-source code. Requests to access the dataset earlier should be directed to zofia.wrona.dokt@pw.edu.pl.

## Abbreviations

IoT	Internet of Things
ECC	Edge–Cloud Continuum
SLA	Service Level Agreement
NAB	Numenta Anomaly Benchmark
PEWMA	Probabilistic Exponential Moving Average
HiTL	Human in The Loop
IE	Infrastructure Element
AWBS	Adaptive Window-Based Sampling
UDASA	User-Driven Adaptive Sampling Algorithm
QoS	Quality of Service
MAD	Median Absolute Deviation
AdaM	Adaptive Monitoring Framework
MAPE	Mean Absolute Percentage Error
SR	Sample Ratio
JPM	Joint-Performance Metric
